# Measuring system resilience through a comparison of information- and flow-based network analyses

**DOI:** 10.1038/s41598-024-66654-1

**Published:** 2024-07-16

**Authors:** Graham Hyde, Brian D. Fath, Hannah Zoller

**Affiliations:** 1 Department of Physics, Astronomy and Geosciences, Towson University, Towson, MD 21252 USA; 2 Department of Biological Sciences, Towson University, Towson, MD 21252 USA; 3 Advancing Systems Analysis Program, International Institute for Applied Systems Analysis, 2361 Laxenburg, Austria; 4 GFZ German Research Centre for Geosciences, Telegrafenberg, 14473 Potsdam, Germany

**Keywords:** Ecological modelling, Ecological networks, Theoretical ecology

## Abstract

Quantifying the properties of complex, self-organizing systems is increasingly important for understanding the development and state of modern systems. Case studies have recommended sustainability frameworks predominately in literature, but little emphasis has been placed on methodological evaluation. Data availability is often an obstacle that constrains conventional flow-based network analysis, but a novel information-based technique (QtAC) developed by zu Castell and Schrenk overcomes these constraints by modelling interactions between agents as information transfers. This study compares the QtAC method to conventional flow analysis by applying both to the same 90-year dataset containing socio-economic data from the island of Samothraki, Greece. Resilience indicators, based on Ulanowicz’s ascendency analysis, are derived on both the information- and flow-based networks. We observe that the resulting dynamics of the information-based networks align closer with complex system dynamics as theorized by the adaptive cycle model. Additionally, we discuss how QtAC offers different interpretations of network indicators when compared to usual interpretations of flow analysis. Ultimately, QtAC is shown to provide an alternative for complex systems analysis if the data situation does not allow for conventional flow-analysis. Furthermore, we show that the combination of both approaches can yield valuable new insights.

## Introduction

The ability to understand a complex, self-organizing system’s behavior is valuable for the purpose of improving the system’s overall health, resilience, and functionality. Early efforts to model the dynamics of complex systems originated from an ecological perspective since natural ecosystems self-regulate remarkably well, which is why humans often turn to nature for innovative ideas on sustainable patterns^1,2^. The framework of ecological network analysis (ENA) has been extended to model socio-economic systems to evaluate their health and create more sustainable structures. This sustainability-oriented research has amounted in a large body of literature aimed at providing evidence for more sustainable systems through the application of different network analyses within General Systems Science; studies contributing to evidence building are typically context-specific case studies or theoretical advancements [see^3–6^]. While network analysis applications to real-world systems will advance General Systems Science knowledge and enhance the field’s understanding of complex, self-organizing systems’ behaviors, network analysis methodology should also be studied. This work contributes to the field’s ability to understand these systems by evaluating the methodology and results of a recently developed network analysis method based on the exchange of information between system components when applied to a longitudinal socio-economic dataset^3^.

The novel information-based network analysis that this work performs and evaluates is referred to as the “QtAC” method (QtAC standing for Quantifying the Adaptive Cycle) and was developed by zu Castell and Schrenk to create a more standardized and generic method for executing network analysis on a wider range of systems^7^. Effectively, the method overcomes data availability challenges inherent to conventional network analysis and enables users to study systems with fewer data requirements. More precisely, QtAC uses time series of abundance data of systems’ components to estimate dynamic interaction networks, which then provide the basis for further analyses. In this study, the QtAC method is compared to conventional material (or energy) flow-based network analysis, which most of the literature to date has employed. Therefore, there are two parallel network analyses of the same type (specifically, ascendency analysis, an offshoot of ENA) applied to the same socio-economic dataset/system. A thorough discussion of the differences and similarities between information- and flow-based network analysis is given from the quantitative results of this study. More importantly, additional insights stemming from the novel QtAC method are also detailed here.

Ecological network analysis is a method for evaluating internal system interactions and investigating emergent holistic properties, especially those arising from indirect effects. More specifically, it is a mathematical tool for analyzing energy or material flows within a system and quantifying the system’s characteristics; many measures have arisen from ENA such as the Finn cycling index, network synergism, network amplification, and more to describe system properties and ultimately facilitate the diagnosis of a system’s state^3,8^. Overall, ENA is a robust analytical tool that was developed so that scientists could quantitatively describe complex systems with hundreds or thousands of species or functional groups comprising a food web. Quantifying the flows of resources that form the structure of an ecosystem can be a difficult task due to the large numbers of interactions that must be considered. Often, the appropriate data required to fully describe a system are unavailable or demand processing to create a homogeneous dataset. This data availability challenge is what the QtAC method overcomes—the method allows users to work with heterogeneous time series datasets as long as the data reflect outcomes of pairwise interactions, making it an attractive alternative for investigating system behavior.

As stated above, this study utilizes ascendency analysis, an offshoot analysis of ENA that, itself, has foundations in information theory. Ascendency analysis provides three properties that quantify complex, self-organizing systems’ universal behavior and they provide information about how interconnected a system is, how capable it is to develop, and how capable it is to persist in time, respectively. These include (1) total system capacity to develop, (2) ascendency, a measure of system order and efficiency, and (3) system reserves also referred to as redundancy or diversity, respectively. Research into ecological networks revealed that systems regularly obtain a balance between two opposing system characteristics, ascendency and redundancy^5,9^. Another key concept in this literature is resilience. However, the definition for resilience varies and one of the goals of this work is to shed light on the QtAC method’s novel technique for calculating this parameter. zu Castell and Schrenk’s resilience measure is defined within the context of network science and was inspired by a definition used in Gunderson and Holling’s “adaptive cycle”^7,10^. Gunderson and Holling define resilience as “the magnitude of disturbance that can be absorbed before the system changes the variables and processes that control behavior”^10^. In the context of the phase space plots found in Sect. "Modeling system evolution: the adaptive cycle", a disturbance may cause a system to change adaptive cycle phases, and the inability of a system to navigate all phases implies the system is not resilient to the disturbance it felt. The system variables in this sense are connectedness (ascendency) and potential (capacity to develop), which will be discussed in detail, and the processes are the system’s compartments.

**Figure 1:**
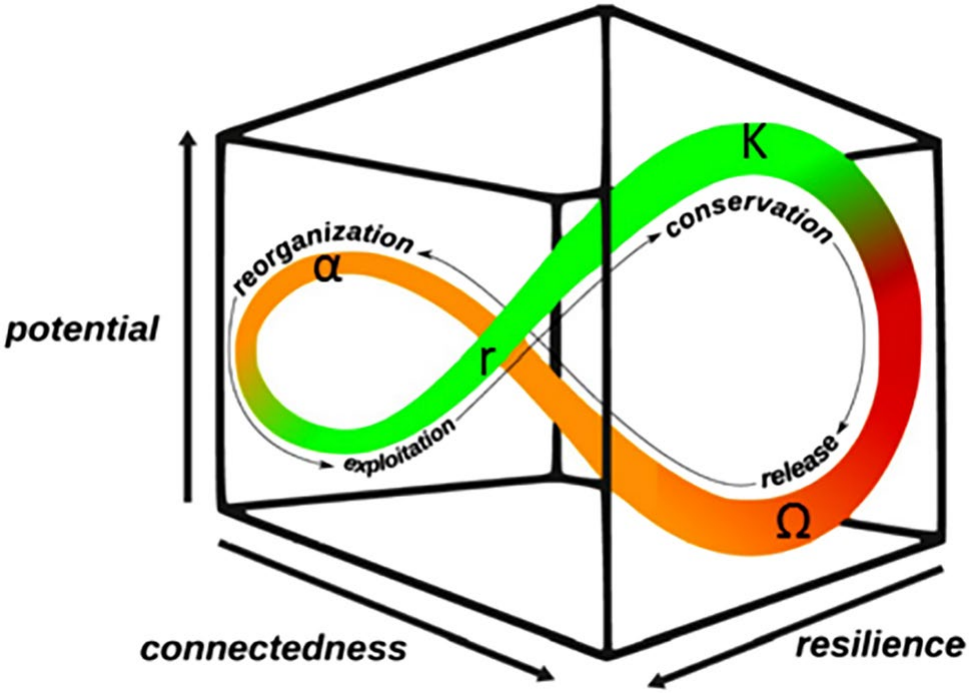
The adaptive cycle model’s four phases depicted in three-dimensional phase space ^7^. Note, the growth phase (r) is referred to as exploitation here—this is a synonymous term.

In 1986, Holling theorized a model called the adaptive cycle that describes the dynamics of complex, self-organizing systems with four phases: growth (*r*), conservation (*K*), release ($\Omega$), and reorientation ($\alpha$)^11^. The adaptive cycle is depicted in three-dimensional phase space in Fig. 1, where the axes represent measurable systematic properties.

When a system is evolving from the *r*- to *K*-phase (growing and developing), the system’s internal structure is becoming more efficient and consequently more rigid, leading to a decrease in resilience. The late *K*-phase is characterized by high system-vulnerability to internal or external perturbations. Conversely, when the system is moving from the $\Omega$- to $\alpha$-phase (collapsing and reorienting), the rigidity of the system’s structure is dissolving and resilience intuitively increases. The system must use its available reserves and structural memory to reorient itself which in turn increases its resilience, enabling the system to enter a new adaptive cycle. With the start of a new *r*-phase and the iterative development of internal structure, resilience begins decreasing again. The adaptive cycle metaphor was a qualitative approach to modeling the dynamics of complex systems observed in nature, and this metaphor has since been extended by Gunderson and Holling to a more abstract, socio-economic perspective where industrial ecosystems (e.g., a city or national economy) are conceptualized to have their own socio-economic “metabolism” like natural ecosystems^10,12–14^. The theories of ENA, ascendency analysis, and the adaptive cycle metaphor have formed a foundation as robust analytical techniques that can be generalized and applied to any complex, self-organizing system, making them useful tools for quantifying a system’s properties and describing its state. While these tools began with a purpose of providing useful information about the state of an ecosystem and what factors are influencing the state, the purpose has been extended to gaining information that can be used to create solutions that improve a system’s health and functionality.

Ascendency analysis and ENA typically use “flow values” to quantify the exchange of resources between two system compartments (here, this is referred to as conventional flow analysis). These flow values have units of whatever resource is being exchanged, for example Joules or kilotons of carbon per time. But for these types of systems analysis techniques, a single currency is required so that the dataset is homogenous which demands the scientist to format the data to a common unit before carrying out any analysis. zu Castell and Schrenk recently developed a standardized technique for performing ascendency analysis where datasets may be heterogenous^7^. The user’s potentially heterogenous dataset is converted to represent transfers of information (nats) using zu Castell and Schrenk’s method, making this technique applicable to any complex, self-organizing system since an exchange of resources between any two compartments will fundamentally lead to an exchange of information. It should be noted that this study employs the concept of information *transfer* as opposed to information *flow*, where information transfer (or predictive transfer) quantifies the amount of information a source node adds to the future state of a target node, thereby reducing the uncertainty in the target node’s future state^15^. Information flow describes the causal effect that the act of changing a source node’s state has on the future state of the target node^15^. The distinction between these two concepts has been made to avoid confusion, and moving forward the concept of information transfer will be used to characterize network properties.

The study presented here quantifies the dynamics of a case study system, Samothraki, Greece, using both information- and conventional flow-based ascendency analysis to model the system’s evolution and investigate how the results of the two analyses compare when applied to the same system. Specifically, the types of results and insights to be gained from the information-based QtAC method in comparison to its flow-based analog are evaluated to understand how and why the analyses represent the same system differently. The implications of the differences between the two ascendency analyses will also be discussed. The choice of this case study was partially motivated by a robust existing dataset available from a previous study by Zisopoulos et al. that quantified the socio-economic metabolism of Samothraki; this dataset contains annual flows of material between thirteen compartments comprising Samothraki’s socio-economic structure from years 1929–2019^3^. Also, the island of Samothraki has been a system of interest in literature recently due to its natural biodiversity and isolated topology. The island is hypothetically more sensitive to import and export flows entering and exiting the island relative to inland countries not surrounded by large bodies of water. If particularly important pathways that connect Samothraki to the outside world are cut off (e.g., shipping routes bringing import materials are eliminated for some period of time), then the system’s network will likely suffer or be less capable of recovering in comparison to inland countries. Additionally, environmental pressures stemming from infrastructure modernization have damaged the island’s biodiversity, motivating recent studies aiming to improve the sustainability of Samothraki. Connecting all these reasons to study Samothraki’s network structure is the general knowledge contribution to network science and its understanding of complex systems’ sustainability. In addition to these research goals, this work is one of the first formal comparisons between information- and conventional flow-based network analysis.

## Information-theoretic ascendency analysis

**Figure 2:**
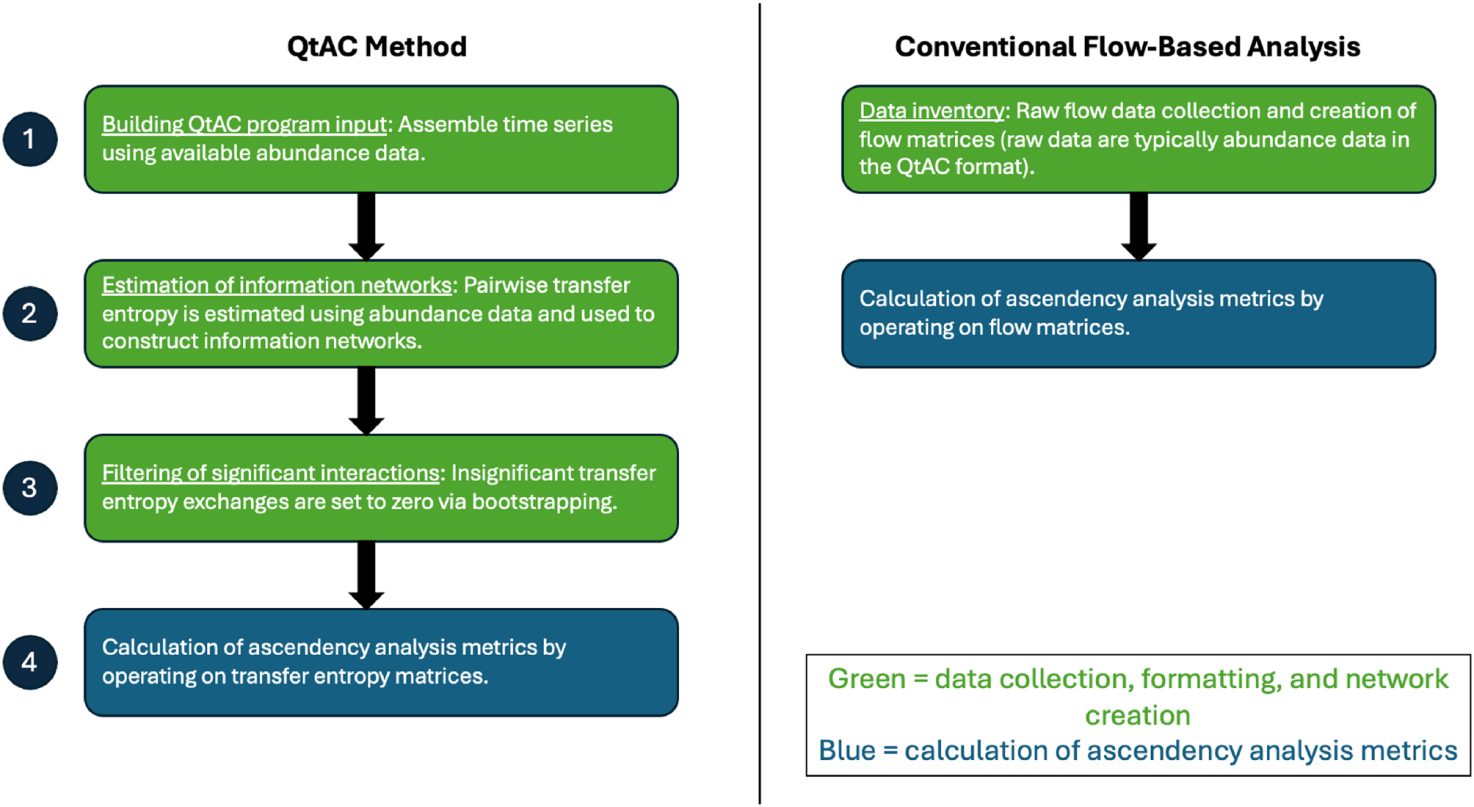
Diagram of how each respective form of ascendency analysis is executed. Note that the QtAC method requires addition steps (and additional computation) before the final information network is obtained and operated on.

Zu Castell and Schrenk’s QtAC technique was published in 2020 as a standardized method of (1) determining the information structure (as a more universal alternative to flow structure) of a general complex, self-organizing system, and (2) computing the system’s position within the adaptive cycle as a means of advancing systems analysis research at large^7^. The QtAC method has an associated R programming package that is accessible in Schrenk’s repository [see^16^]; this R package was used for executing network analyses in this study. It is novel for using information, specifically Schreiber’s transfer entropy, as the quantifying interaction in network analysis in addition to a new approach for calculating resilience, which pulls from spectral graph theory^7,17^. There are two general steps in the QtAC method for calculating the ascendency, capacity to develop, and resilience of the complex system under consideration: (1) estimating the system’s information network structure using Schreiber’s transfer entropy and (2) computing the three ascendency analysis parameters using the information networks. Figure 2 illustrates the general steps to executing ascendency analysis for both the QtAC method and conventional flow-based analysis.

### Estimating information network structures

In order to construct the network structure, we first call upon information theory as follows. Let a complex, self-organizing system be defined as $\nu$ and let each of its system components be represented as stationary discrete Markov process *X* = *(…, X*_*i*_*, X*_*i*+*1*_*, …)* where *X*_*i*_ denotes the random variable underlying the state of *X* at time step *i*. For this study’s Samothraki system, there are thirteen system components (see Zisopoulos et al.^3^ and Noll et al.^4^) and thereby the QtAC method models the Samothraki system $\nu$ with thirteen stationary discrete Markov processes^3,4^. The input data for the QtAC method must be formatted as a longitudinal dataset comprised of data reflecting the outcomes of interactions between the system’s components. In this study, these interaction data are the annual total compartmental material throughflows (in kilotons) of each compartment. This corresponds to an input matrix where columns represent time steps, rows represent system components, and matrix elements contain numeric values representing interaction outcomes for a given time step. An interaction outcome is any general quantity that can describe the result of a system compartment interacting with one or more different compartments. Typically, this interaction represents an exchange of material or energy with other system compartments, but it can take other forms, such as the abundance of plant species (see zu Castell and Schrenk^7^). For example, a matrix element in this study’s time series input represents the total amount of material entering a system compartment (e.g., electricity usage) during a given year. The joint probability densities of all *X*_*i*_ are required to calculate pairwise transfers of entropy for a time step *t*. These densities are estimated on the basis of data within a shifting time window of adjustable size $\omega$, including data between time step *t* $-$ $\omega$ + 1 and *t*. Thus, the method yields a dynamic interaction network from time step 23 onwards. In the current study, a window size of 23 is propagated over the 90-year dataset. The reason for why a window size of 23 is chosen is that this $\omega$ value produced the most stable output. Seeking stable output by tuning program parameters are discussed next, but for a detailed explanation and example of determining stable QtAC parameters, see the corresponding supplementary file. Ultimately, too small of a window size will lead to highly stochastic output curves, and too large of a window will lead to underfitting. Adjusting the window size between these two extremes will eventually produce stable output, where *optimal stability* is loosely defined as smooth output curves with distinguishable trends that are most equipped for users’ analysis goals. A strong indication that a user has reached optimal stability is when slight changes in window size and embedding lengths (*k* and *l* parameters are discussed next) no longer introduce strong trends or patterns in the ascendency analysis results.

A Kraskov-Stögbauer-Grassberger kernel density estimator is used to accommodate small sample sizes and improves upon mutual information estimator algorithms by utilizing k-nearest neighbors rather than binning for assigning weights to local data to estimate transfer entropy^18^. The “destination” component (i.e., the component gaining transfer entropy) is defined to have a Markov order *k* and the “source” component is given Markov order *l*; parameters *k* and *l* represent the embedding lengths of the system’s processes and are adjustable by the user. The size of ${\omega }_{t}$ is left to the user’s understanding of the system and will depend on both how large the input dataset is and the period in which the system’s underlying processes can be assumed to be stationary. Formally, the window size is bounded by the system’s Markov order (all components in the Samothraki system are assumed to have the same Markov order because there is no reason to believe certain components should have different Markov orders) and the total number of time steps^19^. Thus, ${\omega }_{t}$ must fall in the range $\text{max}\left\{k, l\right\}+1\le {\omega }_{t}\le N$^19^. Altogether, there are three tunable parameters for capturing the probability densities (window size ${\omega }_{t}$ and Markov orders *k*, *l*) that reflect the user’s system behavior, introducing a methodological tradeoff between output stability and accuracy in modelling the system’s true dynamics.

After the longitudinal dataset’s probability densities have been estimated, pairwise transfers of entropy between components can be calculated for each time step. Probability densities are necessary because transfer entropy between two components depends on the joint probabilities of components’ present states, conditioned on their past states. The QtAC program estimates a hypothetical information network for each time step where the system components are nodes and the weights of the directed edges are the computed transfer entropy values; estimation results, and subsequently the information networks’ properties, are dependent on user-defined parameters. Because these networks are constructed by *estimated* probability densities that are dependent on user-defined parameters, the QtAC program’s information networks are consequently hypothetical; varying window sizes and Markov orders can lead to a different network structure. The stability of the program’s output should be tested across a certain parameter range by the user. Specifically, the window size, embedding lengths, and significance factor parameters built into the QtAC program should be varied during stability tests. Each time step’s information network is thereby a graph described by Eq. (1)^20^.

1 $${G}^{t}=\left(\nu , \left\{{T}_{Y\to X}^{t}|(Y,X)\in \nu \times \nu \right\}\right)$$

The graph ${G}^{t}$ consists of nodes representing the components of the complex system $\nu$ and directed edges whose weight is given by the respective transfer entropy (units of nats). Transfer entropy will be detailed next in Sect. "Schreiber’s transfer entropy".

### Schreiber’s transfer entropy

Schreiber derived transfer entropy to account for the inability of time-delayed mutual information to filter out information shared between the histories of two interacting system actors^20^. Transfer entropy improves this metric by conditioning transition probabilities^20^. It describes the amount of influence one system component has on another’s transition probabilities and fully accounts for the direction of information transfer. The equation of transfer entropy takes a form similar to that of conditional mutual information. Mutual information can be constructed from Shannon entropy, a fundamental quantity that is also used to derive the general measures of ascendency analysis. Shannon entropy (${H}_{X}$) of a random variable *X* over an alphabet ${\alpha }_{x}$ represents the average amount of surprise, or information contained in an event (equivalently, a realization of *X*), and it takes the form ${H}_{X}=-\sum_{x\in {\alpha }_{X}}p\left(x\right)\text{ log}\left(p(x)\right)$. Conditional entropy ${H}_{X|Y}$ quantifies the amount of information required to quantify the average amount of surprise in *X* given the realization of *Y* is known.

$${H}_{X|Y}=-\sum_{y\in {\alpha }_{Y}}\sum_{x\in {\alpha }_{X}}p\left(x,y\right)\text{ log}\left(p(x|y)\right)$$

Mutual information (*I*_*X;Y*_) quantifies the average reduction in uncertainty about the outcome of *X* given the outcome of *Y* is known. It is related to ${H}_{X|Y}$ by *I*_*X*;*Y*_ = *H*_*X*_ − *H*_*X*|*Y*_ = *H*_*Y*_ − *H*_*Y*|*X*_ = *I*_*Y*;*X*_, hence the symmetric nature of the measure. Consider the case where the destination and source variables *X* and *Y*, respectively, gain information from another discrete random variable *Z*^20^. Conditional mutual information symmetrically measures the expected reduction in uncertainty in the outcomes of *X* and *Y* given the outcome of *Z* is known.

$${I}_{X;Y|Z}=\sum_{z\in {\alpha }_{Z}}\sum_{x\in {\alpha }_{X}}\sum_{y\in {\alpha }_{Y}}p\left(x,y,z\right) \text{log}\left(\frac{p(x|y,z)}{p(x|z)}\right)$$

Schreiber’s transfer entropy transforms ${I}_{X;Y|Z}$ into a dynamical measure (it takes into account the temporal aspect of information transport) by first considering the random variables *X* and *Y* as stationary discrete Markov processes where *X* = (*…, X*_*i*_*, X*_*i*+*1*_*, …*) and *Y* = (*…, Y*_*i,*_* Y*_*i*+*1*_*, …*) with finite orders *k* and *l*, respectively. The Markov order of a process refers to the number of preceding states in a process’s history that are needed to predict the current state of that process; a fixed order model is used for both *k* and *l* throughout this study. If the Markov property below holds, then the state of *Y* has no effect on the transition probabilities of *X:*

$$p\left({x}_{i+1}|{x}_{i}^{(k)}\right)=p\left({x}_{i+1}|{x}_{i}^{(k)},{y}_{i}^{(l)}\right)$$

Considering the Markov property previously defined and the respective Markov orders *k* and *l* of two stationary discrete Markov processes *X* = (*…, X*_*i*_*, X*_*i*+*1*_*, …*) and *Y* = (*…, Y*_*i,*_* Y*_*i*+*1*_*, …*), entropy transferred from *Y* to *X* is defined as

2 $${T}_{Y\to X}=\sum_{{\alpha }_{{X}_{i+1}}}\sum_{{\alpha }_{{Y}_{i}^{(l)}}}\sum_{{\alpha }_{{X}_{i}^{(k)}}}p\left({x}_{i+1},{x}_{i}^{(k)},{y}_{i}^{(l)}\right) \text{log}\left(\frac{p({x}_{i+1}|{x}_{i}^{(k)},{y}_{i}^{(l)})}{p({x}_{i+1}|{x}_{i}^{(k)})}\right)$$

Equation 2 quantifies the average amount of information a source node (the source node being *Y* as Eq. (2) is written) contains regarding the future state of a destination node which itself does not contain in its past^20^. Conceptually, transfer entropy effectively measures the reduction of uncertainty in the future state of a destination node given its past states as well as the source node’s past states. Note that Eq. (2) requires numeric values for the probabilities associated with the outcomes of three variables and this is why probability density estimation is the first step in the QtAC method, a step not taken in conventional flow analysis. In practice, estimating the probability densities and transfer entropies in each time step is a heavily computational process. zu Castell and Schrenk’s QtAC R package uses Lizier’s JIDT toolkit, an open-source, Java-based information-theoretic package, to perform these beginning steps^21^.

### Ascendency analysis on information networks

The three measures of conventional ascendency analysis—ascendency (*A*), capacity to develop (*C*), and reserves ($\Phi$) —are described with intercompartmental flows (material or energy), where it’s typical to represent a flow from compartment *i* to compartment *j* with the notation *T*_*ij*_. Total compartmental throughflows are written as *T*_*i.*_ and *T*_*.j*_ where the dot notation represents a summation over an index. Subsequently, *T*_*i.*_ and *T*_*.j*_ represent numeric values for the sum of all flows exiting node *i* and the sum of all flows entering node *j*, respectively. Furthermore, ${T}_{..}$ represents the total system throughflow (a summation of all flows present in a network) for a given time step. Ascendency (*A*) measures the amount of order in a network structure, i.e., the ability of the network to channel resources between nodes efficiently. In conventional flow-based ascendency analysis, the counterpart of ascendency is reserves ($\Phi$), which represent a network’s lacunae, or unutilized redundancy in its structure that functions as a buffer to perturbations. These two measures add together to quantify the system’s capacity to develop: $C=A+\Phi$. The three ascendency analysis measures in conventional flow-analysis are shown in Eqs. (3), (4) and (5).

3 $$C=-\sum_{i,j}{T}_{ij}\text{log}\left(\frac{{T}_{ij}}{{T}_{..}}\right)$$

4 $$A=\sum_{i,j}{T}_{ij}\text{log}\left(\frac{{T}_{ij}{T}_{..}}{{T}_{i.}{T}_{.j}}\right)$$

5 $$\Phi =-\sum_{i,j}{T}_{ij}\text{log}\left(\frac{{T}_{ij}^{2}}{{T}_{i.}{T}_{.j}}\right)$$

The QtAC method differs from conventional flow-based analysis by computing another measure, resilience, rather than reserves. As mentioned, resilience has taken various meanings in the literature and the QtAC method’s definition relies on spectral graph theory while quantifying Gunderson and Holling’s definition of resilience^10,17^; it will be referred to simply as resilience, but going forward it is assumed to be the QtAC method’s definition. In the QtAC context, resilience quantifies a system’s ability to absorb perturbations and persist in time. This form of resilience cannot be directly derived from $A$ and $C$ and has origins more closely related to the concept of graph connectivity. Still, it will be shown that the novel method’s resilience measure is indirectly related to reserves. In order to transform these equations to information-based ascendency analysis, throughflows *T*_*ij*_ are replaced by transfer entropies ${T}_{I\to J}^{t}$ and the measures now take the forms:

6 $${C}^{t}=\sum_{(I,J)\in \nu \times \nu }{T}_{I\to J}^{t} {\text{log}}_{2}\left(\frac{{T}_{I\to J}^{t}{T}_{..}^{t}}{{T}_{I}^{out,t}{T}_{J}^{out,t}}\right)$$

7 $${P}^{t}=-\sum_{(I,J)\in \nu \times \nu }{T}_{I\to J}^{t} {\text{log}}_{2}\left(\frac{{T}_{I\to J}^{t}}{{T}_{..}^{t}}\right)$$

To distinguish between conventional flow analysis and the QtAC method’s information theoretic framework, ascendency and capacity to develop have been denoted as ${C}^{t}$ and ${P}^{t}$, respectively; these are referred to as connectedness (ascendency) and potential (capacity to develop), respectively, in the QtAC framework. For comprehensibility, the results for both conventional flow-based and information-based analyses will be denoted by connectedness and potential (reserves and resilience are different quantities, so theses will retain their respective names). The QtAC method models a complex system as a mathematical graph in a stricter sense than conventional flow-analysis and zu Castell and Schrenk suitably use connectivity from spectral graph theory to quantify resilience^17,20^. The connectivity of a graph is essentially how readily a new component can be made in the graph by losing edges. Creating a component through a perturbation to the system means to break the existing network, which in the context of the adaptive cycle means to exit the $\Omega$-phase and never reorient, at least in the original unified shape and identify of the system. This measure only applies to undirected graphs, so to apply it to directed graphs the in- and out-degrees of each node are considered. The in- and out-degree matrices, ${D}_{in}$ and ${D}_{out}$, must be calculated for directed graphs in order to calculate the graphs’ Laplacian matrices, which are defined as

$${L}_{out}=c\cdot {D}_{out}^{-\frac{1}{2}}\left({D}_{out}-A\right), {L}_{in}=c\cdot \left({D}_{in}-A\right){D}_{in}^{-\frac{1}{2}}$$

where *c* is a standardization constant, and *A* is the graph’s weighted adjacency matrix. The standardization constant c for each time step is taken to be the reciprocal of the greatest transfer entropy value,

$$c=\frac{1}{\text{max}\left\{{T}_{I\to J}^{t}|(I,J)\in \nu \times \nu \right\}}$$

Users may choose other standardization options that exist within the QtAC method and associated R package (see Schrenk et al.^22^), but this study employs the standardization option (“max weight”) presented above. The smallest real part (${\sigma }_{G}$) of the set of nontrivial eigenvalues of the Laplacian matrices’ unified spectra ($\text{Spec}\left({L}_{out}\right)\cup \text{Spec}\left({L}_{in}\right)$) represents the connectivity of the graph; if all eigenvalues are trivial, the resilience is set to zero. If ${\sigma }_{G}$ is close to zero, only a small perturbation is required to breakdown the structure of the network. However, if ${\sigma }_{G}$ is much greater than zero, a greater perturbation is needed to break the system from its adaptive cycle, implying a greater ${\sigma }_{G}$ value represents a graph with greater resilience. The final information-based ascendency analysis measure, resilience, is represented in Eq. (8).

8 $${R}^{t}=\text{min}\left\{\left|\mathfrak{R}{\sigma }_{G}\right|: {\sigma }_{G}\in \text{Spec}\left({L}_{out}\right)\cup \text{Spec}\left({L}_{in}\right),{\sigma }_{G}\ne 0\right\}$$

## Comparison of results: information- and flow-based network analysis

The origin of where the two forms of ascendency analysis differ will first be discussed in this section. It will be shown that they model system interactions in different ways which demands a different and separate interpretation when applying the information-theoretic results to other network indicators within the realm of ENA. Two network indicators, total system throughflow (*TST*) and Finn Cycling Index (*FCI*), will be used to demonstrate the challenge of calculating network properties using the QtAC method’s output.

### Reasons for discrepancy

**Figure 3:**
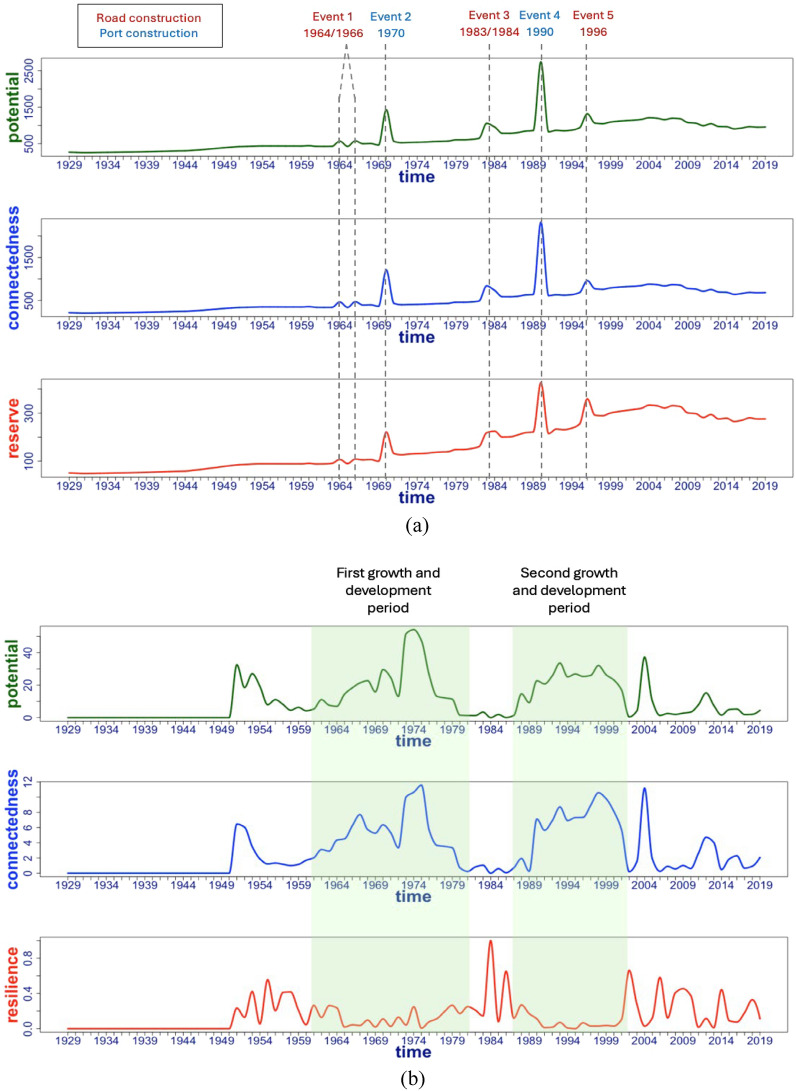
Comparison of flow- and information-based ascendency analyses from the Samothraki dataset: (**a**) Flow-based results (units: kt material) with significant socio-economic events labeled, and (**b**) information-based (QtAC method) results (units: bits) with major growth and development periods labeled.

The temporal trends of the ascendency analysis metrics (capacity to develop, ascendency, and reserve/resilience) of the information- and material flow-based analyses are shown in Fig. 3 and clearly differ. Note that the first 22 years in Fig. 3b are set to zero because the transfer entropy data are absent—a window size of 23 was used in the QtAC method to estimate transfer entropies, yielding results from 1951 onwards. Years 1929–1950 in Fig. 3b are set to zero for easier comparison between the two methods and are not results of this study’s system analysis.

The dataset used in this study, available from Zisopoulos et al., has a material flow matrix for each year from 1929 to 2019^3^. These conventional flow matrices represent the annual flow networks that contain information about which compartments (or nodes) are exchanging material and how much material is being exchanged. To the researcher’s furthest knowledge, the material flow matrices represent the system’s truest network structure, where the truest structure in this context is taken to be the material flows that create the actual pathways that define the network under investigation. The conventional flow-based analysis will produce the truest representation of Samothraki’s network structure simply because it identifies the exact pairwise interactions from the beginning, and then operates on those flow matrices to obtain the three ascendency analysis metrics. Ascendency analysis is a functional analysis, meaning its measures are dependent on the network’s connections and magnitudes of exchanges. Therefore, conventional flow analysis (results in Fig. 3a) contains all aspects needed to completely carry out ascendency analysis once the material flow matrices are identified and filled: Conventional flow matrices have both *directionality* and *magnitudes*.

The material flow results in Fig. 3a capture notable socio-economic events, many of which are identified in Noll et al. and Fischer-Kowalski et al.^4,23^. Each small and large peak in Fig. 3a is associated with either a port or road construction that demanded atypically large amounts of material. The general increasing trend in Fig. 3a is attributable not only the growth of physical infrastructure, but to farming subsidies that effectively required more materials for agricultural practices and growing livestock populations^4,23^. In the 2000s, the trend plateaus in response to removed subsidies, the shutdown of diesel generators, and financial crises^4,23^. Altogether, the material flow results show that the confluence of infusing materials and economic opportunities into the system, and constructing physical pathways for trade had an impact on the island’s potential, connectedness, and reserves. The island is forming or enhancing pathways for material to flow, and the additional resources are transformed to reserves, providing the island with a greater capacity to develop. It has been shown that material flow-based results detected materially demanding events in the past 90 years of Samothraki’s socio-economic history, proving the ability of conventional ascendency analysis to identify system’s real-world socio-economic patterns when relevant data are available. But why is conventional material flow analysis seemingly better at capturing historical socio-economic activity compared to the QtAC method’s analysis?

While the network’s structure is already known in conventional flow analysis, it has to be *estimated* from a time series of abundance data reflecting the outcomes of system interactions. Consequently, the QtAC method must take the additional step of probability density estimation before calculating the ascendency analysis metrics. The method begins with input data that do not have pairwise directionality given the format of abundance data, meaning only the magnitudes of a timestep’s collective interactions are known. Specifically, the abundance data of this dataset reflect the total amount of material flowing through each given node in single time step, which reflects the effective magnitude of interactions each node experienced. The user must make the judgement that the abundance data capture effective system interactions at an appropriate scale for a stable analysis. Only once probability densities in each shifted window have been computed through the kernel density estimator can pairwise transfer entropies be calculated for each timestep and directionality inferred. The two network properties needed to complete ascendency analysis are now known—directionality and magnitudes of flows (in this case, “flows” are the transfers of entropy)—resulting in transfer entropy matrices for each time step that appear to be equivalent to, or an information analog to conventional material flow matrices. However, the transfer entropy matrices from the QtAC program are not conceptually equivalent to the conventional flow matrices and this is the fundamental difference between the two forms of ascendency analysis. Notably, the transfer entropy networks are much sparser than the conventional flow ones as described below.

While the material flow matrices from conventional flow analysis define the specific exchanges from one system compartment to another and thereby model the concrete flow structure of the Samothraki system, the QtAC method is modeling *dependencies* between compartments. Transfer entropy measures the information being transferred from a source node’s past to a destination node’s future, which has not already been contained in the destination node’s own past. Equation 2 indicates transfer entropy is dependent on conditional probabilities—how the future state of one node depends on its current and past states as well as the source node’s current and past states. If the abundance of process *X* depends on the abundance of process *Y*, then there will be a nonzero matrix element. Conversely, if the abundance of *X* (i.e., the transition probabilities of *X*) do not depend on *Y*, there will be a zero matrix element. Therefore, transfer entropy matrices estimated by the QtAC method represent the pairwise dependencies of all system processes for each time step, effectively modeling the temporal development of the Samothraki system’s dependencies. This is different than simply modeling the exchange of some quantity of material between system processes.

One can also see that conventional flow-based analysis is more capable of detecting notable events or system perturbations at specific time points in a dataset. The QtAC method’s results do not articulate these events as well as the conventional analysis. Instead, the information-based results show gradual extrema surrounding notable events and perturbations since they model system actors altering their *dependencies* with one another rather than the tangible annual flows of material. Because conventional flow-based analysis models more concrete, directly measurable flows (flows of material rather than flows of information) between system compartments, the conventional form of ascendency analysis is more reliable for analyzing system behavior at single time steps. The QtAC method, modeling the less tangible flow of information, does not appear reliable for analyzing system behavior at single time steps—it provides insights into system behavior over chunked *intervals*. The resolution of these intervals will depend on the user’s chosen input data, dataset size, and final judgement of results. Despite this drawback, the QtAC method adds the additional layer of quantifying dependencies between compartments, which will have implications when we discuss total system throughflow (*TST*) in Sect. "Applications to other network indicators".

In conventional flow analysis, a nonzero matrix element in the initial flow matrices may not necessarily represent a dependency between the two interacting processes. Figure 3 shows that transfer entropy and material flow are not equivalent. In other words, there is no proportionality constant between the two exchange media in this case study. The dependency data from the QtAC method are capturing an inherently different system response to changes over time. The two prominent periods in Fig. 3b where potential (capacity to develop) and connectedness (ascendency) rise and fall coincide with significant socio-economic changes in Samothraki. During the 1960s–1970s and the 1990s, the island obtains subsidies that effectively increase agricultural output and economic activity at large, and ports and roads are constructed to support increasing imports, exports, and infrastructure. Intuitively, it appears that the system’s compartments become increasingly dependent on each other during periods of atypical activity. The compartments view the socio-economic changes as perturbations and respond by effectively “communicating” more, or more accurately, increasing their dependencies on one another to respond to change. Information-based network analysis is modeling the system through a different perspective despite executing the same equations as conventional flow analysis. The blatant difference between the two forms of ascendency analysis is a modelling choice left to the user: Is one interested in how system compartments depend on each other or how they exchange materials/energy? The former choice investigates a more fundamental relationship between system actors while the latter investigates more direct exchanges within a system that define the network.

Finally, one may question the considerable window size ($\omega$ = 23) required to estimate probability densities for the Samothraki system. While the output’s stability was optimized with this window size, the first 22 years of the dataset were excluded from ascendency analysis, meaning system behavior could not be viewed from 1929 to 1950. This is a tradeoff that the user makes when using the QtAC method: The lower data requirements (i.e., any type of time series data can hypothetically be used to study system behavior) make this analysis less restrictive, permitting several or many system analyses to be carried out, but some data must be expended in probability density estimation. In general, the window size of a dataset that reaches optimal stability will vary between studies and it is context dependent. For example, zu Castell and Schrenk studied the development of a plant community in a prairie-forest in Eastern Kansas^7^. Their dataset consisted of 14 time steps and they used a window size $\omega$ = 6, leaving an ascendency analysis of nine years^19^. Within this small timeframe, they were able to identify a full adaptive cycle (the system navigated all four phases), the effects of intentional burns at the beginning of some years, and explain the general behavior of plant species using information networks^7^. Thus, while it is inevitable that some input data will be expended on initializing the transfer entropy estimations, system behavior will still be quantifiable and observable. The degree of detail in output is left to the user’s judgement and requirements for analysis.

### Disconnected networks in the transfer entropy framework

As mentioned, the existence of a material or energy flow in conventional flow analysis does not necessitate a dependency, i.e. a nonzero transfer entropy between two nodes in the context of the QtAC method. Significant amounts of transfer entropy exist in the Samothraki networks during time periods when socio-economic changes (e.g., port construction) are occurring, implying the transfer entropy networks will be sparser during socio-economically dormant time periods. When the Samothraki network is in a more static state, there is less aggregate surprisal in the network since there are no perturbations (or perturbations of lesser magnitude) affecting the abundances (abundance referring to the amount of material passing through a compartment) of the system compartments, leading to less information existing in the network. Without changes in the compartmental abundances of the system, there is a lack of information since it is generated from the amount of surprisal associated with pairwise events. Thus, unlike conventional flow analysis where materials or energy are always present in the system, there is a possibility for transfer entropy networks to be disconnected if the abundances, or the states, of the system compartments remain relatively constant. Results generally showed that Samothraki’s information networks became sparser when they exhibited higher resilience, which may appear counterintuitive. However, the strength of the few (or only) node edges (compartmental pathways) in the sparse networks were typically greater than the weakest edges in denser networks, resulting in this phenomenon where sparser networks appear more resilient.

**Figure 4:**
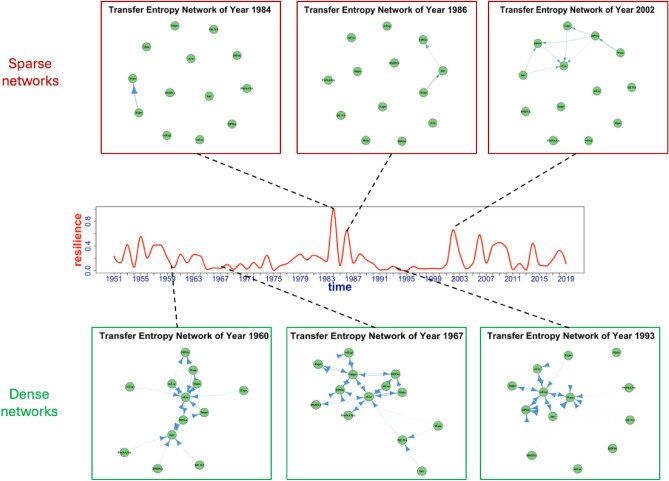
Examples of disconnected and connected information networks from the QtAC method’s results. Sparsest networks commonly occur during years of greatest resilience for the Samothraki network. Denser, more connected networks generally occur during years of lesser resilience.

**Figure 5:**
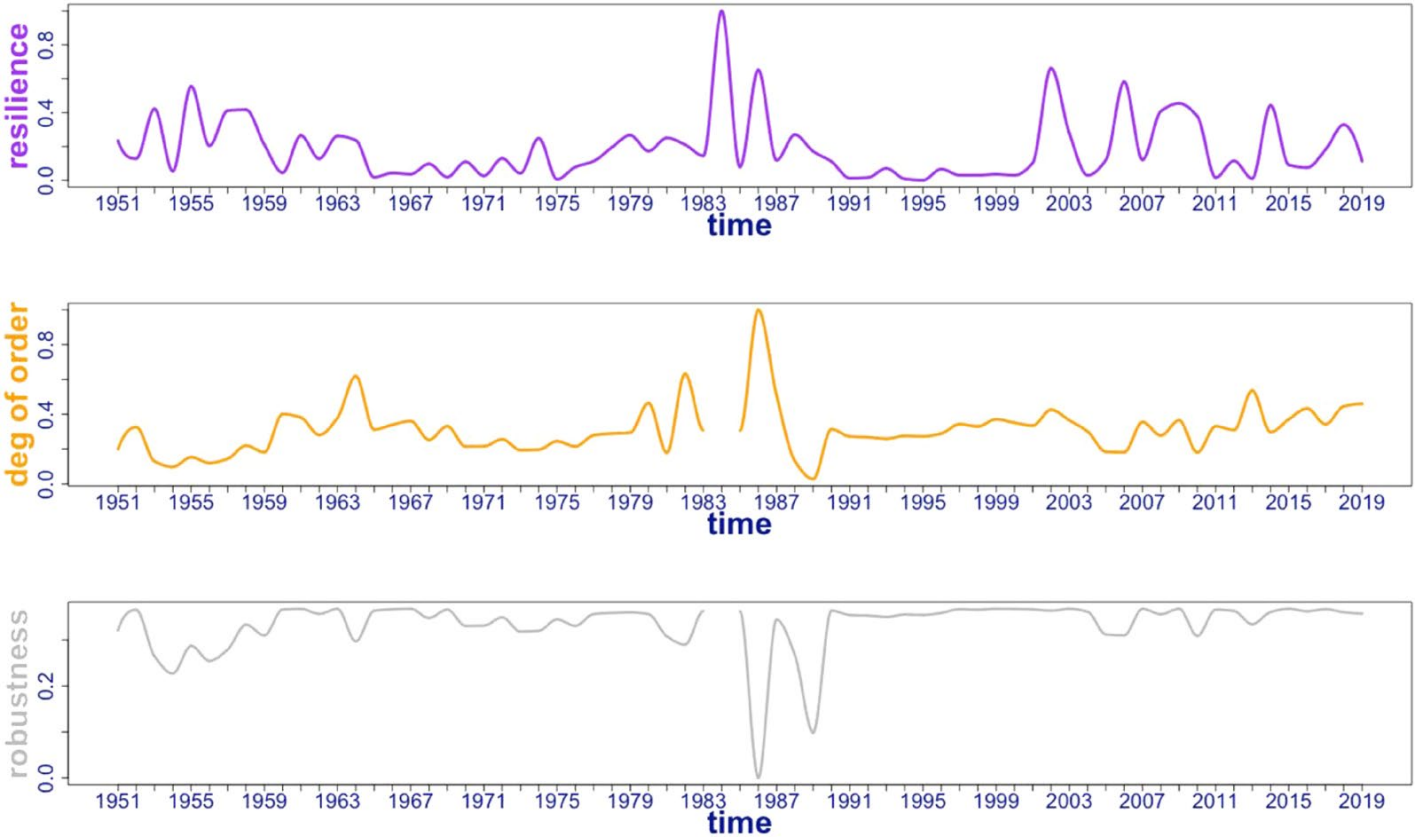
QtAC resilience is the purple curve, the degree of order ($\alpha$) is the yellow curve, and robustness (*r*) is the grey curve. Note that $\alpha$ and *r* are undefined during year 1984 because capacity to develop is zero. Units of plots are in bits.

The QtAC method treats the networks in Fig. 4 by only considering the connected nodes, effectively applying ascendency analysis to each time step’s connected subgraph. The connectivity of these subgraphs happened to be greater than the connectivity of all 13-node graphs in the entire set of QtAC results, which may seem counterintuitive from the conventional flow-analysis perspective since one of its fundamental requirements to begin analysis is a connected network (graph). *Robustness*, *r* = $-\alpha \text{ln})$ (where the *degree of order* $\alpha =\frac{A}{C}$), is a metric used in conventional ascendency analysis that quantifies the general sustainability, or fitness of the system to survive^5^. The degree of order quantifies the amount of efficiency, or organization within a network structure, hence the name “degree of order”. Calculations of Samothraki’s degree of order using transfer entropy data show that $\alpha$ and resilience have similar behavior. This can be explained from the analysis of the information networks’ sparsity (Fig. 4) where highly resilient networks contained few dominant pathways, leading to high degrees of order, or efficiency. As we’ll discuss, these disconnected graphs cannot make physical sense, but $\alpha$ has proven to be a valuable metric for analyzing complex systems’ organizational behavior and achieved sustainability; for example, $\alpha$ has been used in architecting processes in system of systems (SoS) research^24^. Robustness is sometimes associated with a system’s resilience, which refers to the confusion in terminology mentioned in Sect. "Introduction: demand for generalizable network analysis techniques". Most natural ecosystems fall within a small interval over $\alpha$ where robustness is maximized, and this interval has been deemed the “window of vitality”^5^. This has motivated researchers to quantify socio-economic systems using robustness to compare them to natural ecosystems (the best performing self-organizing systems). Using the QtAC method’s output for this study, robustness was calculated and compared to connectivity-based resilience in Fig. 5.

Figure 5 shows that robustness and connectivity-based resilience are displaying opposite behaviors—where resilience is maximized in the 1980s, robustness experiences its greatest minima in the entire the time series. The discontinuity in the $\alpha$ and *r* time series comes from the division by zero since ascendency is zero in year 1984. From the robustness perspective, Samothraki’s degree of order grows in the 1980s and then returns to an average value that it fluctuates around. While not many insights can be gained from this metric (which has been calculated from transfer entropy data), it clearly exhibits inverse behavior relative to connectivity-based resilience. The inability to decipher which metric aligns with the system’s events introduces limitations to QtAC resilience. Users must consider these differences when evaluating results. Perhaps one of these two sustainability-oriented metrics may align with the true physical instantiation of the system based on historical context and insights become accessible. Neither metric in this study provides valuable insights into Samothraki’s overall sustainability, though the resilience metric does align well with the adaptive cycle model as we’ll see in Sect. "Modeling system evolution: the adaptive cycle". We find that the QtAC method’s resilience successfully captures the phase-oriented behavior predicted by Gunderson and Holling, an interesting phenomenon that deserves attention. Subsequently, Sect. "Modeling system evolution: the adaptive cycle" will be discussing the information-based results’ ability to model the adaptive cycle and connectivity-based resilience will be used throughout.

In this case study, all thirteen compartments must be connected in some way (both weakly and strongly connected graphs meet this requirement). If the sparse networks of Fig. 4 were material flow networks, the network data would be incomplete, and the system model would have to shrink to exclude all disconnected compartments. In other words, the disconnected networks of Fig. 4 do not satisfy conventional ascendency analysis requirements and the analyses would not begin if the exchange medium were materials or energy. However, the QtAC method still computes the ascendency analysis for each time step despite the presence of disconnected graphs. The method does this by considering only the connected subgraph that contains nonzero edge weights and applying the ascendency analysis equations (Eqs. 6 and 7) along with the resilience equation (Eq. 8) to the subgraph. With transfer entropy as the exchange medium, a disconnected network may be permitted since an unchanging system may produce no surprisal and subsequently no information.

Because the QtAC method’s networks may be disconnected, this is another significant difference in the beginning stages of these two forms of ascendency analysis. It is theoretically possible to have sparse and disconnected transfer entropy networks if the system is unchanging, which can occur in real socio-economic systems, as shown here with Samothraki. However, this difference makes direct comparisons between material-flow and information analysis even more difficult since the system model is changing in the QtAC results while remaining the same (out of necessity) in material-flow results.

### Applications to other network indicators

**Figure 6:**
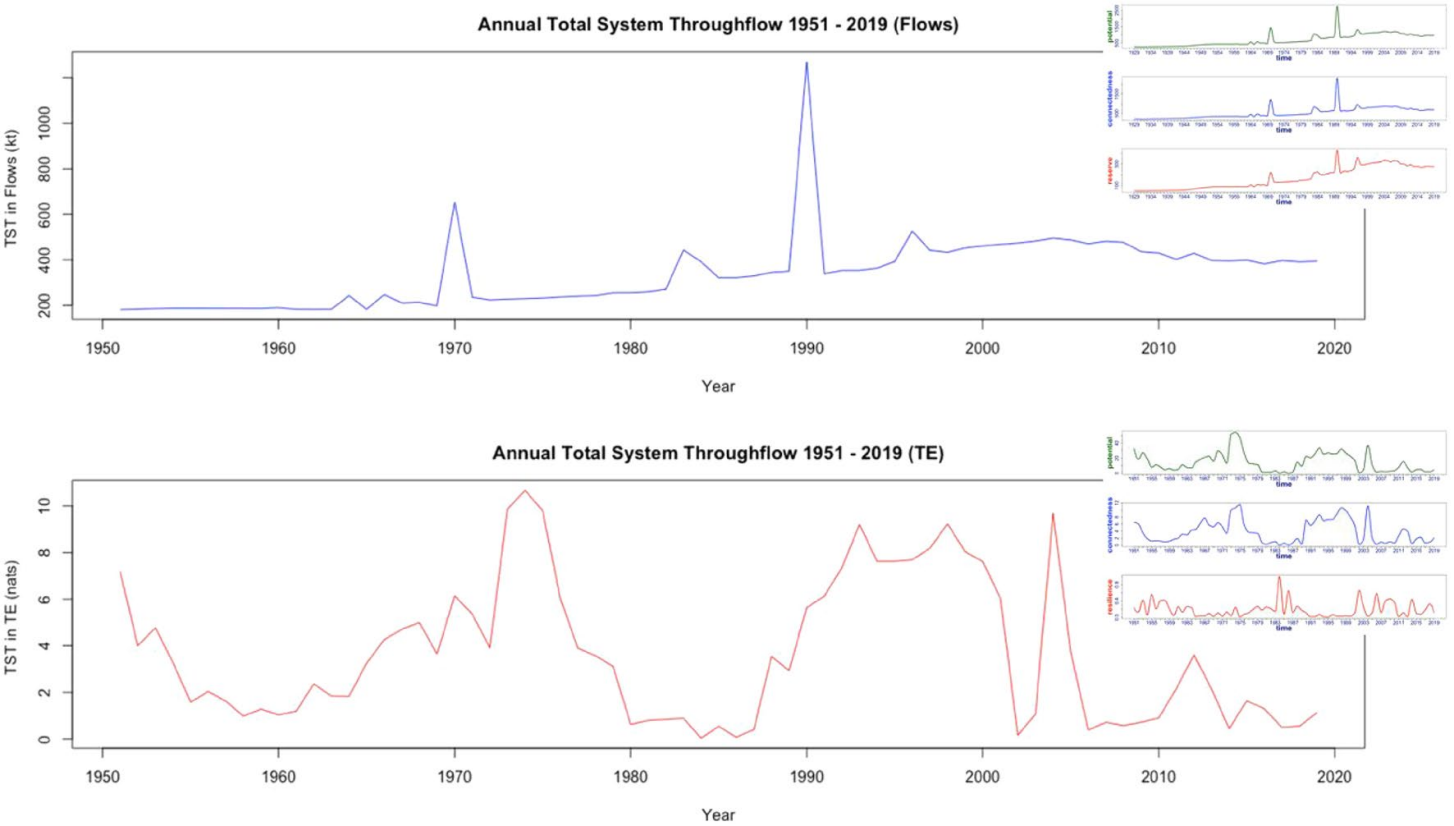
TST of the Samothraki system from years 1951–2019. The top and bottom plots represent the TST in terms of kilotons of material and nats, respectively. Ascendency analysis curves from each respective analysis are included to highlight the similarity in behavior between TST and ascendency analysis measures.

Ascendency analysis measures are scaled by the total amount of material, or in the QtAC method’s case, information, present in the system (generally, the total system throughflow). In terms of material flow analysis, total system throughflow represents the total amount of material the Samothraki system used per year. For the information-based analysis, *TST* represents the total amount of entropy transferred (*TE*) between compartments per year. However, the *TST* metric communicates the same message in both forms of ascendency analysis: how *active* the system is at a given time. Figure 6 shows the *TST* for both the information- and flow-based analyses. Corresponding longitudinal plots from Fig. 3 are attached to show similarities in curve behavior for the two respective analyses.

Because the QtAC method is modeling the dependencies between system actors based on the probabilities of the existences of pairwise relationships, *TST* immediately takes on a new meaning for the information-theoretic framework. *TST* in material flow analysis intuitively indicates how much material the system is using in steady-state whereas *TST* in information analysis indicates how much total information was exchanged within the system in steady-state. This new information-based perspective of *TST* suggests that when more information is present in the Samothraki system at a given time, the system compartments are becoming increasingly dependent on each other in response to atypical system perturbations so that the network can adapt. Transfer entropy data that produce the information-based *TST* plot in Fig. 6 are not directly related to annual material flows within the network, but they imply a deeper meaning that is how the network’s nodal relationships change over time. Thus, the information-based *TST* can be thought of as the Samothraki network’s nodal responses (changes in compartmental dependencies) to the material-based *TST*. This adds a different perspective to the conventional view of *TST* and offers another layer of understanding to systems analyses. Specifically, examining a system’s changing pairwise-dependencies allows users deeper insights into the importance of particular system compartments to the network’s behavior. The trends of the three ascendency analysis measures for both the convention flow-analysis and information analysis in this study are quite similar to their respective *TST* trends. It appears that *TST* drives the ascendency analysis measures, which for a highly linear socio-economic metabolism like Samothraki’s is fitting. This phenomenon is likely to be observed in other linear industrial ecosystem models where resource cycling is minimal.

The QtAC method’s results do not translate as well to the Finn Cycling Index (*FCI*) indicator as they do when applied to *TST. FCI* measures the percentage of cycled flows (*CF*) that exist in a network at a certain point in time; cycled flows originate and terminate at the same compartment. The *FCI* indicator reflects how much material or energy a system is reusing before expelling it from the system boundaries and is thereby a suitable metric for investigating an aspect related to the system’s sustainability^25,26^. The flow matrix containing all *T*_*ij*_ values can be operated on to obtain a dimensionless form that represents each flow value as an intensity relative to the total compartmental throughflow. This flow intensity matrix *G* is determined by dividing each throughflow value of a compartment by its respective total compartmental throughflow so that each element is represented as ${g}_{ij}=\frac{{T}_{ij}}{{T}_{j}}$. We can identify flows that have traveled *m* path lengths before terminating at a compartment by raising the *G* matrix to the degree *m* which can be represented as a series summation.

As the degree *m* approaches infinity, the matrix elements will approach zero and this defines the integral flow matrix *N* where *I* is the identity matrix.

$$N=\left[{n}_{ji}\right]=\sum_{m=0}^{\infty }{G}^{m}={\left(I-G\right)}^{-1}$$

**Figure 7:**
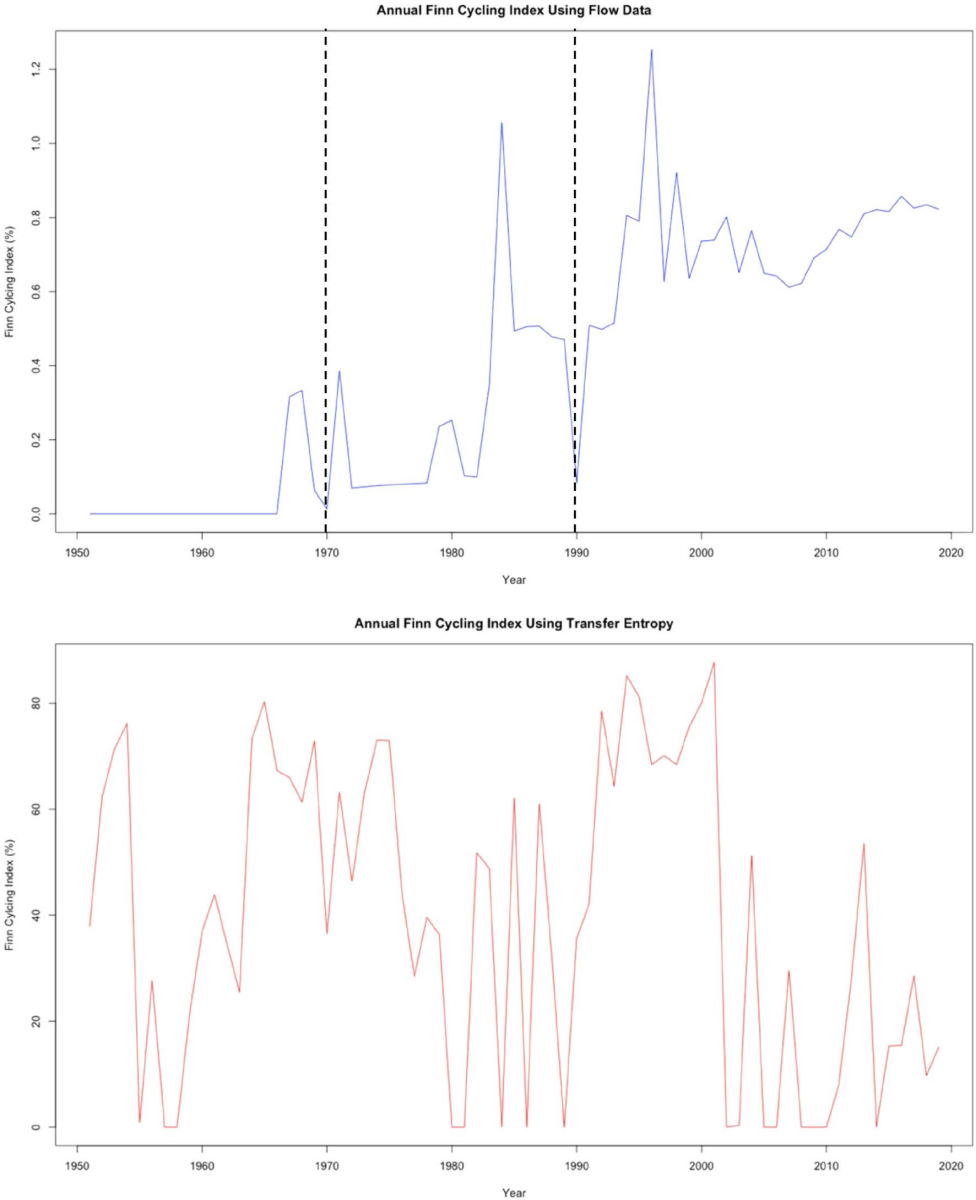
*FCI* for years 1951–2019 for Samothraki’s material flow (top) and information (bottom) networks. Significant minima are labeled in the material flow data that correspond to port constructions.

*FCI* is defined as

9 $$FCI=\frac{\sum_{i}\frac{{n}_{ii}-1}{{n}_{ii}}{T}_{i}}{TST}=\frac{CF}{TST}$$

where *T*_*i*_ represents the total inflow to compartment *i*. Estimated transfer entropy values are substituted for the material flows in Eq. 9. Figure 7 shows the *FCI* percentages over time for both ascendency analyses.

The general overall trend in flow data is for increasing TST. However, the maximum cycling index for material flow data is only about 1.2%, a considerably low value which is common in industrial ecosystem models due to their typical linear flow structures. Some studies on other industrial ecosystem models have found cycling indices less than 1% because of their system structures’ high linearity, meaning they typically use material/energy flows once before expelling those flows from system boundaries without reuse^27,28^. As the system’s *TST* grows over time, the system has more available flows within its network, increasing the probability that some flows will be reused before existing the system. Severe drops in the material flow-based *FCI* occur in 1970 and 1990 when the system imported large amounts of material to support infrastructure constructions, which are socio-economic processes that use resources for one purpose before converting the flows to outgoing waste exports (hence, a process with little cycling). Therefore, this material flow analysis produces *FCI* trends and insights that are reasonable considering Samothraki’s socio-economic activity during 1951–2019. This logical trend in flow cycling breaks down when using transfer entropy to investigate *FCI.*

When using transfer entropy values, the Samothraki system exhibits atypically high *FCI* values that are not reached by natural ecosystems when using transfer entropy values. Natural ecosystem models serve as optimal examples for complex system behavior and have higher *FCI* values than industrial ecosystems due to their unique recycling (detritus) components, making the information network results appear unphysical^29,30^. Surprisingly, maximum and minimum *FCI* percentages of 87.7% and 0%, respectively, are seen. The *FCI* of information networks fluctuate between large values outside of typical natural ecosystem cycling and extremely small values, reaching zero in multiple years. The most unusual aspect in these results is that the system may have a remarkably high *FCI* in one year and a zero *FCI* the subsequent year, showing no sense of gradual change. Socio-economic systems have an inertia that prohibits intense changes in macroscopic variables like the *FCI* on a year-to-year basis, so it would be more sensical to expect system changes due to perturbations spread out gradually over time. More importantly however, how does one interpret a single transfer of entropy *cycling* through multiple system components like a material flow does? It is understandable how a single flow of material may cycle through a network’s nodes, giving an indication of how well the network reuses resources. But the “cycling” of pairwise transfer entropies does not provide any information regarding how sustainable a system is—there does not appear to be a direct relationship between the recycling of physical resources and recycling of information. An understanding of Samothraki’s sustainability is difficult to arrive at through the transfer entropy form of *FCI*, and in this situation, the indicator is not of much use. Here, we have a situation where the QtAC method’s output cannot be translated appropriately to an ENA network property used to characterize systems. While the transfer entropy form of *TST* contained some novel and useful insights into nodal dependencies, the *FCI* represents a case where the information-theoretic framework fails to help us understand the sustainability of the system.

## Modeling system evolution: the adaptive cycle

Plotting ascendency analysis’ potential as a function of connectedness over time allows one to view the phase-oriented development of a system. It is found that the QtAC method’s results show phase-oriented system behavior that is predicted by Holling’s adaptive cycle model (Figs. 6 and 7). Contrary to the transfer entropy results, the material flow data will not produce cyclic behavior predicted by the adaptive cycle model and this is clear by inspection. Hence, analogous plots to Figs. 6 and 7 would show only linear curves, providing no insight into phase-oriented behavior.

**Figure 8:**
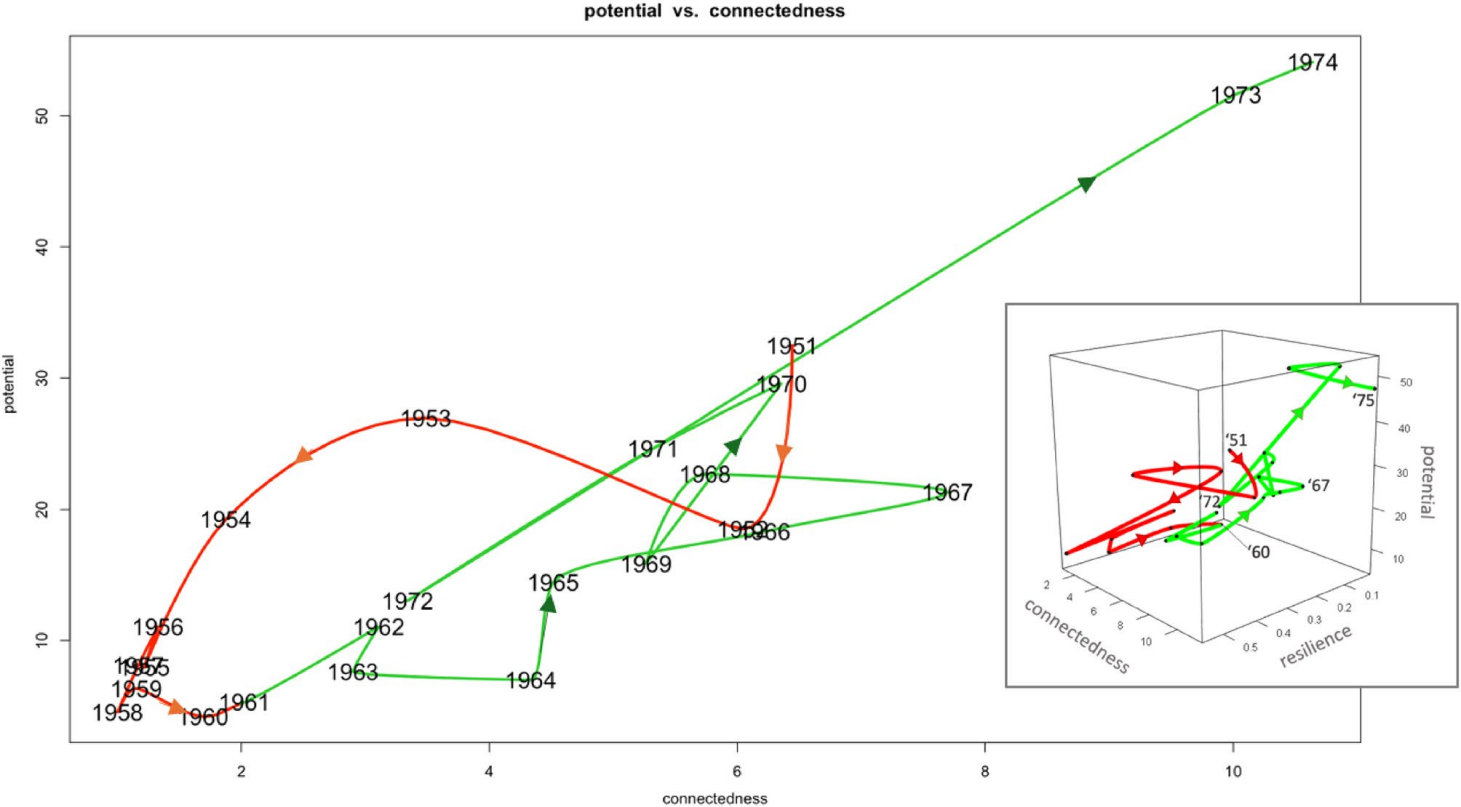
Potential as a function of connectedness for years 1951–1974. Points are labeled by their respective year and the units of the axes are bits. Red curves represent collapse and reorientation phases, and green curves represent growth and conservation phases. The subplot includes the resilience dimension and contains the same potential and connectedness data.

**Figure 9:**
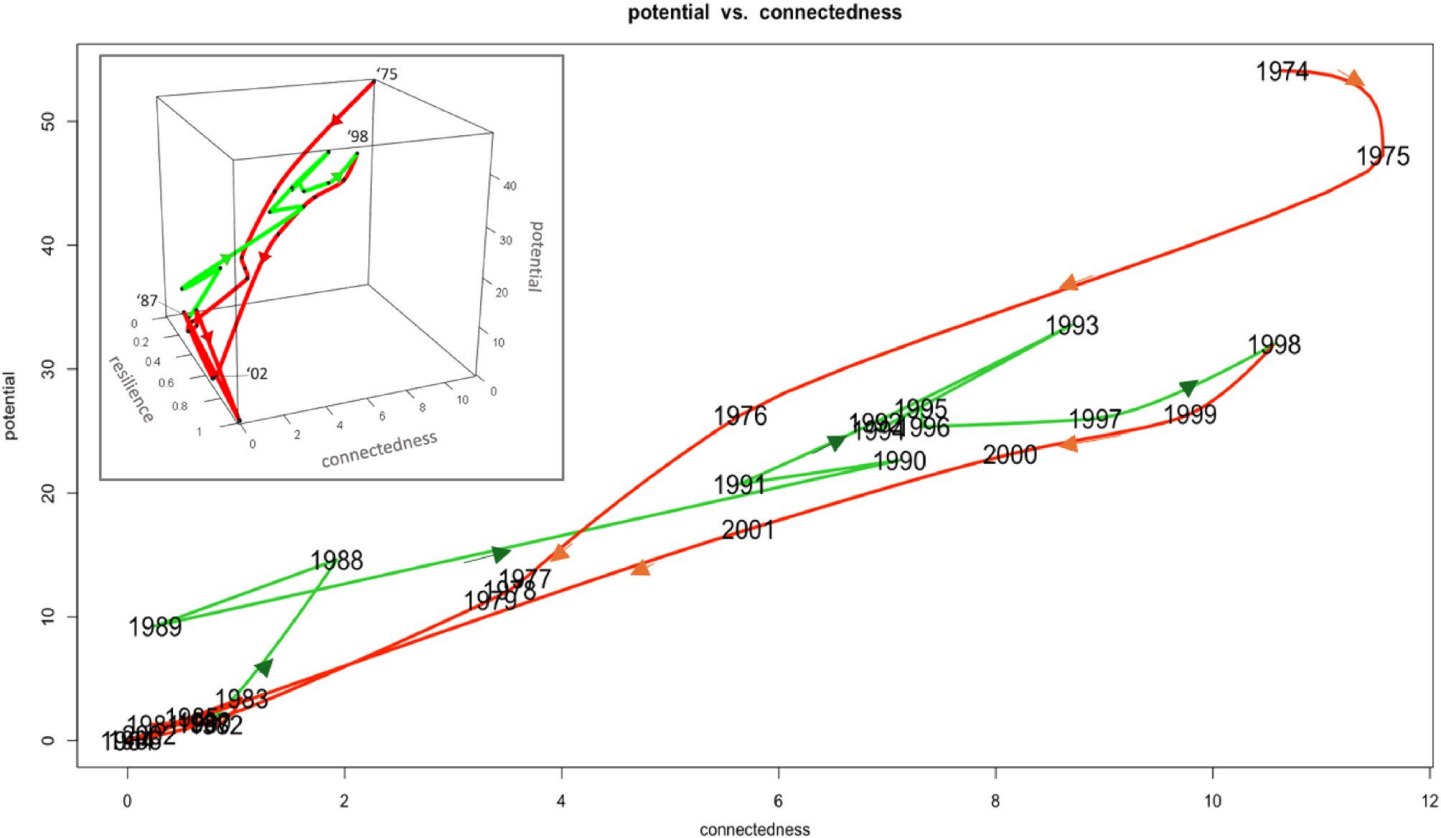
Potential as a function of connectedness for time periods 1974–2002. Coloring notation denotes the same phases as Fig. 8. Yearly data from 1951 to 1973 were removed for clarity. The subplot again includes the resilience dimension and contains the same data depicted in the 2D plot.

Figure 8 begins with the Samothraki system reducing its potential and connectedness from 1951 until its minimum in 1958 and then increasing going into year 1964. Samothraki’s increasing connectedness (ascendency) implies the network’s pathways between system compartments are enhancing, i.e. some pathways are becoming more dominant (magnitude of dependencies are increasing) and important to overall system behavior. In parallel, the Samothraki’s network’s capacity to develop increases. While the system is decreasing these two parameters, resilience increases (see Fig. 3b), indicating system reserves (flexibility and diversity in network connections) are being used to enter a new growth phase. An equivalent interpretation of the usage of reserves is that Samothraki’s network structure is dissolving, leading to lower rigidity and thereby vulnerability. Figure 8 shows the continued growth phase until 1974 with some sub-cycling occurring in years 1967–1972 where Samothraki underwent its first stage of accelerated socio-economic growth (e.g., port construction, construction of an electricity network, state subsidization). Thus, this first long-term cycle indicates Samothraki experienced socio-economic growth and development starting around 1960, causing system compartments to depend on each other the most around 1974. Here the adaptive cycle model has provided phase-oriented insights into the socio-economic conditions of Samothraki as opposed conventional flow-analysis’s insights which only quantify amounts of material or energy. These observations support the predictions of the adaptive cycle that a system undergoes large-scale and small-scale cycling during a growth phase before reaching its conservation phase where potential and connectedness are maximized. Figure 9 continues the phase-oriented system development in time shown in Fig. 8.

Figure 9 captures the second long-term cycle from the QtAC results. Towards the origin of Fig. 9 where potential and connectedness are minimized in the 1980s, the system exhibits high resilience (see Fig. 3b), meaning it is using reserves to recover and enter another growth phase. Almost anticipating the major port extension in 1990 that is visible in the material-flow results (Fig. 3a), the information-based results capture a growth phase starting in the late 1980s where network pathways are again strengthened (greater order and efficiency) and the system’s overall capacity to develop increases. The second long-term growth phase spans 1987–2002 where potential and connectedness reach another maximum in potential and connectedness in 1998 (conservation phase) before collapsing again for the remainder of the time series aside from an outlier event in 2004. This captures the second stage of accelerated socio-economic growth where the island received more subsidies, invested in infrastructure growth and development, and resultantly increased dependencies on imports and exports. Therefore, quantifying the adaptive cycle model of the Samothraki system has shown to shed light on the phase-oriented behavior of the system as it evolves. It captures the growth, development, and breakdown of network structure’s rigidity, i.e. pathways are strengthened, made more synergistic with each other to the benefit of the system’s health, and subsequently weakened in order for future adaptations to occur. As we’ll discuss, the time period following these two long-term cycles displays erratic behavior in terms of potential and connectedness, which verifies the island’s stagnated socio-economic activity entering the 2000s. Admittedly, the phase transitions from growth to conservation and collapse to reorientation cannot be identified with precision since the QtAC method’s results in this study are too unstable to analyze single time steps. On the other hand, transitions from conservation to collapse and reorientation to growth are identifiable since those relate to minima and maxima. Altogether, we conclude that conventional flow-analysis is equipped to give time-step-specific details regarding the amounts of material flowing between particular pathways, but the QtAC program contributes a complementary interpretation of the system’s evolutionary phases of increased order and disorder based on the dependencies between system compartments.

One can see that the QtAC’s resilience measure peaks when potential and connectedness are minimized (see Fig. 3b). This aligns with the three-dimensional theoretical adaptive cycle model—a system is most resilient when moving from a reorientation phase to a growth phase, which is when the system is in an “infancy” stage with low degrees of ascendency and capacity to develop. At this point in phase-space near the origins of Figs. 6 and 7, the system has utilized its untapped reserves ($\Phi$), or its available flexibility to optimize network connections, and it begins collecting activation energy, which in terms of the QtAC method’s information framework, corresponds to the emergence of new transfers of information in the network (or an overall reduction in uncertainty) stemming from external or internal perturbations. It makes sense that the largest peak in resilience occurs in the 1980s because that describes the system’s state between its two significant acceleration phases. In the 1960s and 1970s, the system enhanced its socio-economic infrastructure in ways that positioned it as a more modernized system—it built an electricity network, gained subsidies bringing in more income, and established roads and a port. We see the system has multiple peaks in resilience in the 2000s likely reflecting the turbulent financial situation Greece was experiencing. From an adaptive cycle standpoint, the system undergoes shorter cycles in phase-space which would imply it is experiencing unstable conditions. Therefore, QtAC offers a different interpretation for the resilience property in ascendency analysis. While *reserves* computed from material flow represent the overall redundancy in a network, *resilience* computed from transfer entropy data represents the ability of a network to withstand a perturbation of a particular magnitude. Resilience is thus related to the amount of reserves a system contains, and we have now found a situation where the information-based analysis is dependent on material flow analysis’s results and offers an alternative perspective on a system property.

Why is it that only the information-based analysis shows phase-oriented behavior that aligns with the adaptive cycle? Recall that the QtAC method models the temporal dynamics of dependencies, or, in other words, effective interactions, between system compartments. From the transfer entropy perspective, system compartments are “communicating” more (concretely, the uncertainty in future state of one compartment is being reduced by the knowledge on the state of another compartment) when the system’s capacity to develop and ascendency measures are growing because the system is learning how to optimally organize its structure, requiring alterations in compartmental connections/dependencies. If the system fails to respond to a perturbation or for other reasons cannot retain its current internal structure, it breaks down in the sense of the adaptive cycle. The dynamics of the system compartments’ pairwise dependencies describe how the compartmental *relationships* change, which immediately gives insight into how important or influential certain compartments are relative to others. In material flow analysis, the dynamics only describe which compartments exchanged material annually with each other (where dependencies may or may not exist)—one must perform further calculations to understand nodal importance. Additionally, the QtAC program allows the user to consider the influence of past states on the future state of a system unlike conventional material flow analysis, incorporating more depth into the ascendency analysis. It is reasonable to expect that the QtAC method’s results align with the adaptive cycle model since the model is describing the dynamics of a complex system through time when experiencing change, and more importantly, how the system responds to those changes during its trajectory. Examining how the system compartments adjust their dependencies on each other over time will explain how the internal organizational structure of the system responded to change, whereas examining what material flows existed over time only explains how direct input to the system was distributed throughout the network. Thus, the QtAC method, and more generally the use of transfer entropy to quantify interactions, appears promising in confirming and investigating the theoretical adaptive cycle model.

Loosely speaking, QtAC measures *effective interactions.* By effective, we mean that the interactions between the system components do have an effect on the abundance data being used for the network estimation. Hence, in general, there might be a material flow between two components but no information transfer (if the material flow does not present an *effective* interaction), and, conversely, there might be an information transfer between two components but no corresponding material flow (if the dependency between the two components is an artifact or if it is caused by an interaction different than the measured material flow). Consequently, we cannot expect a one-to-one correspondence. In the current case study, however, flow data and abundance data do have a special relation, since the abundance of a component at a given time is the sum of all in- and outgoing material flows. In this sense, every interaction should be effective, and the only interactions underlying the abundances should be the measured material flows. Still, QtAC yields networks which are very different to the flow networks. One of the reasons surely is the fact that the effects of different interactions (flows) overlay each other in forming the abundances. Besides, dependencies caused by common sources, indirect interactions, etc. might occur as well. The main reason, however, is that information transfer is conceptually different from a material flow. Information transfer can only exist if the abundances of a compartment develop in a way that is not fully explainable by its past development. A system at equilibrium, with abundances being all constant, does not exert any information transfer, no matter how strong the material flows. On the other hand, a system being hit by a perturbation, leading to a cascading loss of material flows, will show an increasing number of (strongly fluctuating) information transfers between its components. This information theoretic view on system behavior aligns much closer with the system development described by the adaptive cycle metaphor: a stable, mature, but vulnerable complex system is characterized by only few connections, while a system breakdown can lead to the emergence of many new connections—characteristics which might be counterintuitive at first.

## Conclusions

This study investigated a novel systems analysis method (QtAC), developed by zu Castell and Schrenk, by applying it to a 90-year dataset (available from Zisopoulos et al.^3^) containing socio-economic data from the island of Samothraki^3,7^. A parallel ascendency analysis was applied to the dataset using conventional material flow analysis to identify differences between the novel and conventional methods and any additional insights that the novel method introduces to ascendency analysis. It was discovered that the two methods will produce vastly different results superficially because they model different types of interactions. Material flow analysis simply models the exchanges of material between system compartments, which defines the observed network structure (i.e., what pathways and the flow magnitudes exist), but information-based analysis models the information transfer between system compartments, leading to a directed, weighted network of dependencies. While the two forms of ascendency analysis differ, it was shown that connections can be made between their respective results in some cases, adding an additional layer of understanding to the overall system analysis.

The *TST* of the information networks is particularly high during times of economic or political changes, forcing the system to react, leading to an increase in information transfer. It was found that information-based *TST* gives insights into the changing dependencies between system compartments whereas flow-based *TST* simply quantifies the total amount of material or energy present in the system. Future studies may investigate the relationship between notable system events and information-based *TST*, verifying that greater levels of compartmental dependencies align with system perturbations. Exploring specific nodal dependencies may also uncover details regarding the importance of specific network pathways to overall system behavior. While a sensible and useful interpretation was made for *TST*, there seems to be an inability to map transfer entropy data to the *FCI* indicator. *FCI* is used to measure the amount of cycling going on within a system to make assertions about the system’s overall sustainability, which is difficult to do when the interactions quantify only the strength of influences nodes have on each other. No additional understanding was contributed to the *FCI* metric when applying transfer entropy data to it, suggesting that the information framework may not translate easily to all ENA indictors or that further steps must be taken. This conjecture can be further verified in future work by using the QtAC method to calculate other ENA indicators.

The QtAC method’s results from the Samothraki analysis confirmed characteristic phase-oriented behavior predicted by the adaptive cycle model. Modeling the dynamics of compartmental dependencies more intimately captures the system’s response to system input or perturbations. The results describe at which points in time the system contained more information, which pairwise relationships strengthened or weakened, and how resilient the system was to perturbations following notable changes or events (e.g., a rapid expansion in physical infrastructure). Those data aspects are enough to capture the adaptive cycle since they contain information about the system’s nodal *responses* to change over time. Material flow analysis does not necessarily contain information about system response at the level the QtAC method does—it only contains information regarding how the network’s structure exists based on what flows exist at a given snapshot in time. The user must infer system responses from material flow data through further network property calculations, such as robustness, average path length, mean nodal degree, etc.^8^. Further case studies could investigate whether the QtAC method confirms the adaptive cycle, and more interestingly, seek relationships between adaptive cycle phases and network indicators. Discovering correlations between ENA indicators and particular adaptive cycle phases would integrate these two theories and enhance the understanding of complex system dynamics.

The large appeal of the QtAC method is that it enables users to execute system analyses with fewer data requirements, especially when data are scarce or unavailable. The data availability challenge is often in obstacle in conventional material/energy flow analysis. While this was not a challenge in this study, the QtAC results showed clear detection of significant system events throughout the dataset, such as road and port constructions, subsidizations, and other historical changes that are viewed as perturbations. Thus, this novel method that utilizes transfer entropy to define network structures seems to be promising as an alternative to conventional flow analysis, especially since it offers different insights into network properties in some cases, such as a system’s *TST*. The two methods could be executed together to produce a more comprehensive systems analysis where one describes the resource flows that define the network’s structure and the other describes how much influence nodes have on one another. From the perspective of users executing these forms of system analysis, conventional flow analysis is effective for identifying superficial material/energy flows that define the network structures under investigation, and information-based analysis is effective for understanding how system actors relate and depend on each other in response to system change, possibly giving deeper insights into system dynamics and evolution. Ultimately, future research should aim to integrate these two innately different forms of ascendency analysis by applying information-based results to other ENA indicators. It is believed a new and insightful understanding of these indicators may arise when translating them to an information framework.

### Supplementary Information

Supplementary Information 1.

Supplementary Information 2.

Supplementary Information 3.

## Data Availability

The annual material flow data for the 90-year dataset can be found in the supplementary material as an Excel file. This Excel file was created and given to us by Filippos K. Zisopoulos. Both an Excel and text file containing this study’s input (abundance) data for the QtAC program can also be retrieved from the supplementary material. Acronyms used to label the Samothraki system compartments in all supplementary material are defined as follows: (DE) domestic extraction, (DMI) domestic material inputs, (PM) processed materials, (eUse) material inputs for energy use, (mUse) material inputs for material use, (Int/Out) Interim outflows, (wExports) exported waste, (DPOe) domestic processed output emissions, (DPOw) domestic processed output waste. All numeric values represent annual total compartmental throughflows with units of kilotons of material; data taken from direct flow matrices constructed by Zisopoulos et al. 2023.
