# Supplementary material for: Measuring system resilience through a comparison of information- and flow-based network analyses
Source: Sci Rep. 2024 Jul 16;14:16451. doi: 10.1038/s41598-024-66654-1 (PMC11252444; doi:10.1038/s41598-024-66654-1)
Supplement: Supplementary file 3 — Supplementary Information 3. [file 41598_2024_66654_MOESM3_ESM.pdf]

SUPPLEMENTARY FILE

MEASURING SYSTEM RESILIENCE THROUGH A COMPARISON OF INFORMATION- AND FLOW-BASED NETWORK ANALYSES

Graham Hyde[1], Brian D. Fath[2,3*], and Hannah Zoller[4]

1. Department of Physics, Astronomy & Geosciences, Towson University, Towson, MD 21252, USA
2. Department of Biological Sciences, Towson University, Towson, MD 21252, USA
3. Advancing Systems Analysis Program, International Institute for Applied Systems Analysis, A-2361 Laxenburg, Austria
4. GFZ German Research Centre for Geosciences, Telegrafenberg, Potsdam, 14473, Germany

DETERMINING WINDOW SIZE (ω_t) AND EMBEDDING LENGTHS (*k/l*) IN THE QTAC METHOD: SAMOTHRAKI EXAMPLE

The parameters that are manually changed that influence the shape and scale of the output are the window size (ω_t) and embedding lengths (k and l). When ω_t is increased, the KSG kernel estimator has more data to estimate probabilities with which would in theory enhance the probability estimates, but also reduce the model's stochasticity, a property inherent to Markov processes; recall, the QtAC method bases its quantitative approach on the assumption that the system actors are Markov processes. Conversely, if ω_t is too small, the model may not accurately capture interactions between the system's processes. This leads us to what this study has taken to be characteristic of output that best captures the Samothraki system's *truest* network structure across the dataset's time domain: When consistent and distinguishable long-term trends are identifiable and shared by multiple parameter combinations (ω_t, *k/l*), the output resembles the system's network to a higher degree of accuracy compared to output that's atypical, unpredictable, or unshared by other parameter combinations. For example, in five different tested window sizes, two long-term periods are observed where the system's capacity to develop and ascendancy gradually rise and fall around the start of the 1960s and late 1980s. One goal of the study is to evaluate the effectiveness of the QtAC method (in comparison to conventional flow-based ascendancy analysis) in identifying events, either internally or externally driven, that have impacted the island's sustainability from 1929 – 2019. These mentioned impacts are quantified via ascendancy analysis and should appear when mapping the measures to the system's adaptive cycle over time. Thus, the output from the QtAC program that's most suitable to this study's goal would ideally show distinguishable and macroscopic trends over the 90-year timespan. Measures should display notable responses that signal a systemic reaction to either an internal or external

perturbation (e.g., a main road on the island being shut down would be received as an internal perturbation to the socio-economic structure). By nature, a country's socio-economic network structure will change at a yearly scale, so the evolutionary drivers should be identifiable at a minimum time resolution of one year. Let's first examine one set of QtAC output shown in Figure 1.

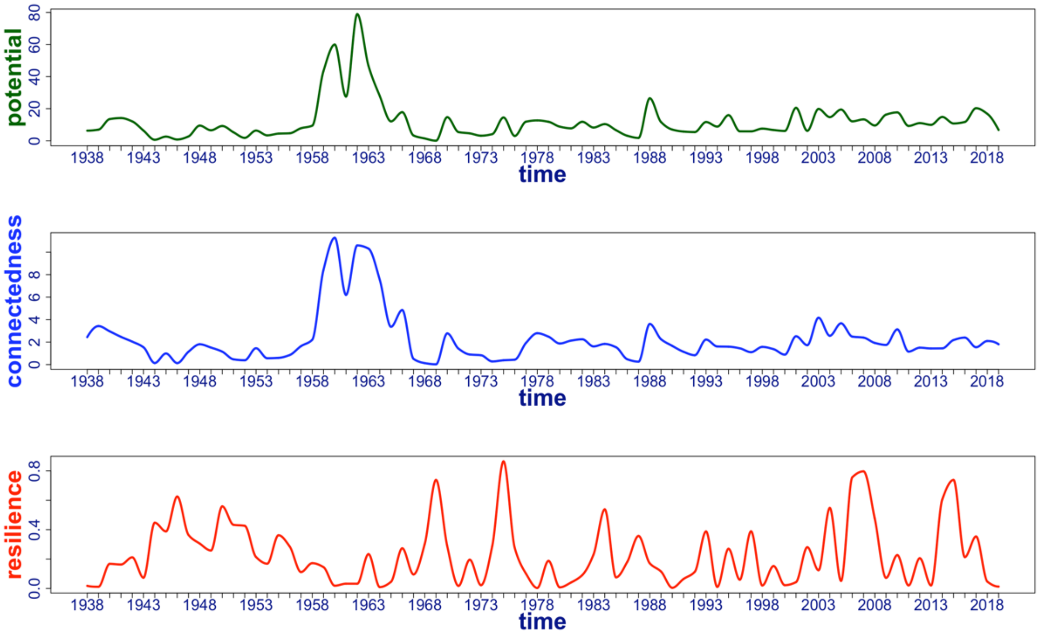

Figure 1: Example output from the QtAC program (ω_t = 10 and *k, l* = 10).

The results above were obtained using a window size ω_t = 10 and embedding lengths *k, l* = 10. Looking at the potential and connectedness curves, we interpret that the Samothraki network became more ordered towards the end of the 1950s where the island began modernizing its economy with the constructions of a port and electricity network. Furthermore, this implies the network self-organized more efficient pathways to channel materials to the system's socio-economic compartments, which is reflected simultaneously through the potential indicator – the system's capacity to develop increases proportionally during these years. Conversely, resilience decreases going into the late 1950s. This is interpreted as the Samothraki system utilizing its activation energy (in the form of "untapped", or available reserves) to enter the growth phase

evident in the other two measures. This growth phase seems to reach a maximum value around the early 1960s that corresponds to an equilibrium phase which is followed by a collapse phase into the late 1960s. However, we notice that the measures show *no distinguishable trends* outside of this one noticeable time period – all trends fluctuate around a relatively constant value, providing no clear or meaningful signal that something interesting has happened. Also, there is no obvious time step where an interesting even has occurred – the results appear stochasitic and noisy. This indicates that the output requires some form of optimization to reveal clearer trends that can be interpreted within the island's historical context. Ultimately, the three ascendancy analysis measures in Figure 1 will represent themselves differently when setting different window sizes and embedding lengths in the QtAC program. Before explaining this study's process of finding optimal program parameters, we start with classifying the types of undesirable output and the consequences of each. This will provide a general outline for how users of the QtAC method can handle any output.

A time window ω_t that is too small will produce highly stochastic results that fluctuate, limiting the user's ability to identify long-term trends. The stochasticity can be reduced by holding the window size constant and increasing the embedding lengths k and l; this increases the number of past states that are allowed to influence the transition probabilities of a process's potential future states. Tests on this study's dataset determined that a window size $\omega_t = 10$ is too small for extrapolating useful trends despite increasing the embedding lengths; datasets of other sizes will follow the same process of evaluating the "smoothness" and distinguishability of events in the QtAC output, but the window sizes will obviously vary. The results would become underfitted before distinguishable trends emerged, implying some information regarding system dynamics is being lost. Figure 2 (a) and (b) show results for embedding length values of k, $l = 4$

and k, $l = 20$, respectively, with a constant window size of $\omega_t = 10$; these combinations were tested arbitrarily. These two test outputs serve to demonstrate this window size's inability to detect stable trends in the dataset.

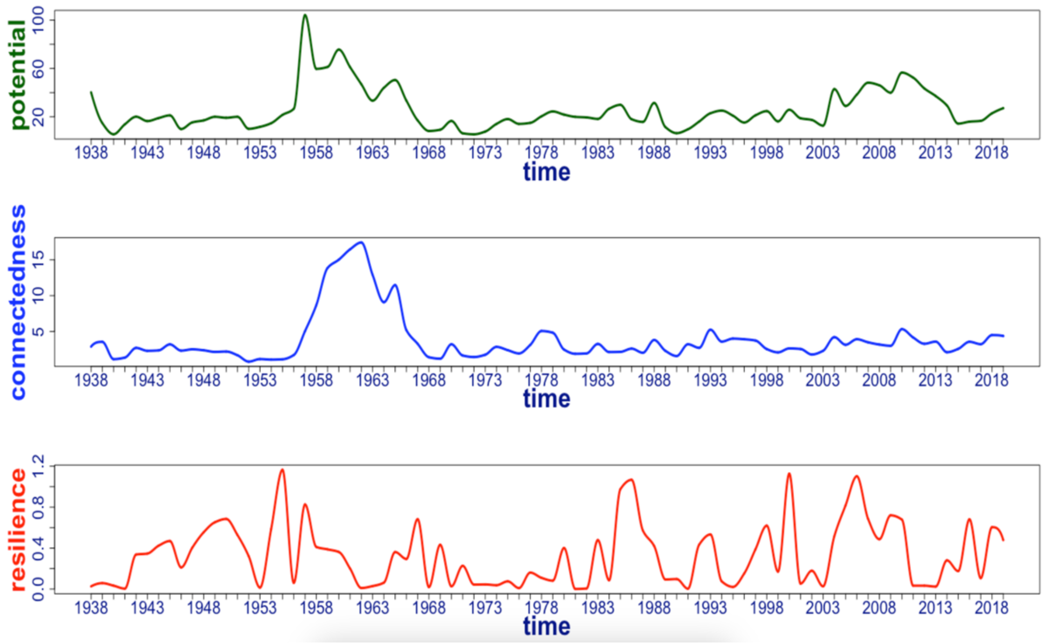

(a)

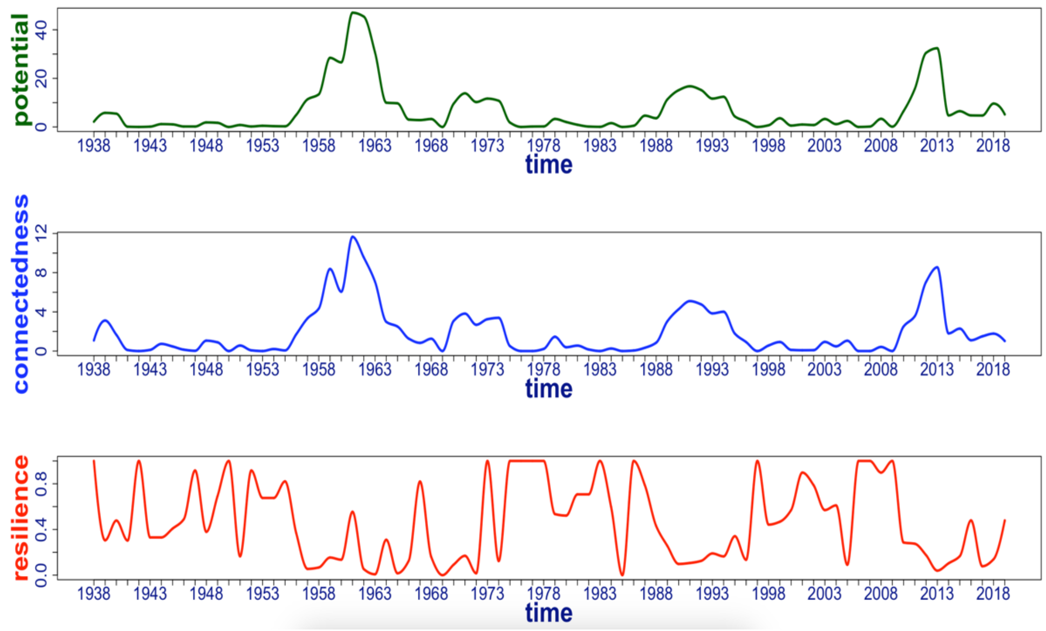

(b)

Figure 2: Visualization of unstable output from the QtAC program. (a) Capacity to develop (potential units: bits), ascendancy (connectedness units: bits), and resilience (units: edges) of the

Samothraki system using ω_t = 10 and k, l = 4. (b) Same results as (a) but using k, l = 20 where underfitting is observed in the curve flattening.

The results in Figure 2 (b) detect some distinguishable patterns that the study's optimal results capture, but the resilience measure shows slight underfitting, especially during the 1970s. Also, both plots are dominated in scale by the maximum that occurs around the early 1960s, indicating that local data within that time window are significant and possibly masking trends later in the time series. Overall, Figure 2 is an example of output that require further parameter tuning.

There are two extremes that the QtAC output can display: over-stochasticity (too much detail or fluctuation) or underfitting (loss of detailed resolution). Figure 3 displays some tests that exhibited behavior from these two extremes. Window size and embedding length combinations of each test shown in Figure 3 (a, b, c) were chosen arbitrarily in this figure to show the extreme output behavior that must be overcome. The optimization process discussed in this section aimed to tune the window size and embedding length values until an appropriate middle-ground between the extremes was found.

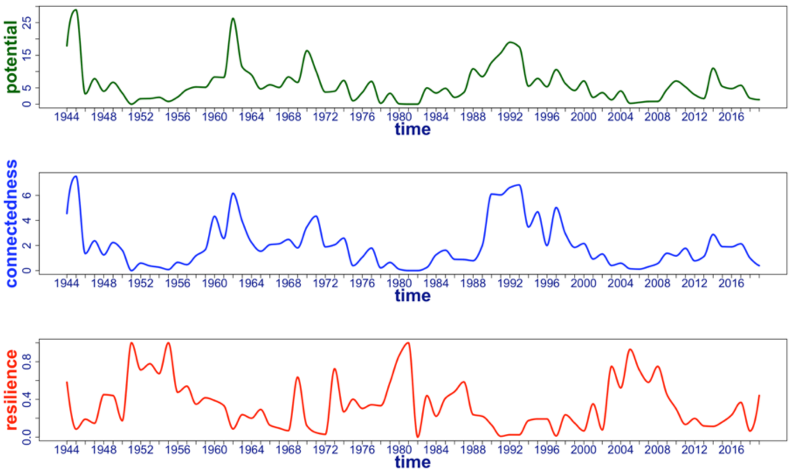

(a)

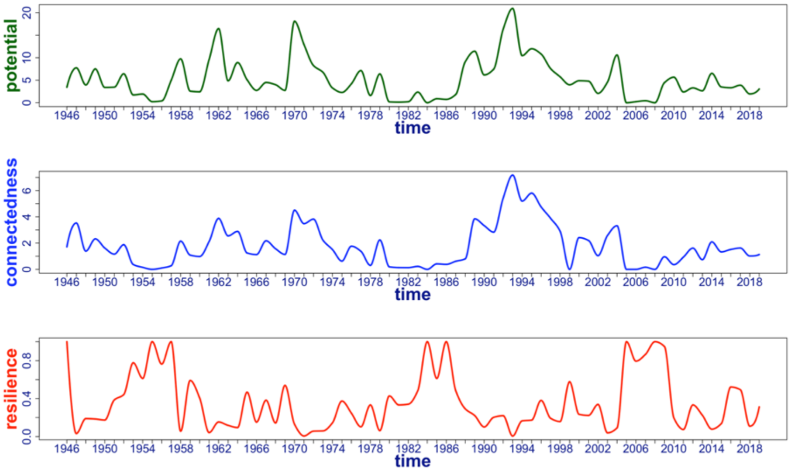

(b)

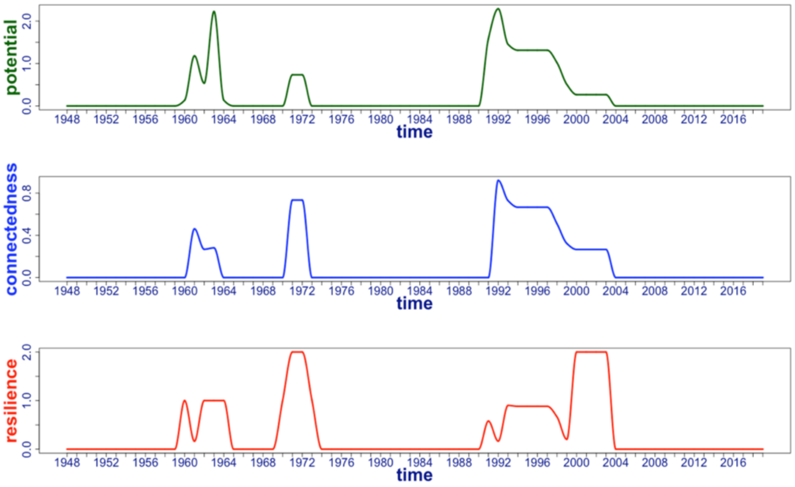

(c)

Figure 3: Visualization of stochasticity and underfitting. Results using parameters (a) ω_t = 16, *k, l* = 9 and (b) ω_t = 18, *k, l* = 11 are too stochastic and unpredictable. Results using parameters (c) ω_t = 20, *k, l* = 25 are underfitted and remove all detail.

One can see the effect of increasing the embedding lengths in Figure 3. Embedding lengths in Figure 3 have increased from those of Figure 2 and more interesting behavior is observed across the time series. Increasing the embedding length accentuates macroscopic trends due to the increased dependence of transition probabilities on past states. Too low of an embedding length will lead to results that are unpredictable, making it difficult to identify system behavior in a localized time period. However, if the embedding length is too high, the measures will saturate because there's too much dependency on each time point's previous states. This saturated behavior is shown in Figure 3 (c). Altogether, this study tested 7 window sizes and many embedding lengths for each window size. Table 1 lists all tested window sizes and corresponding embedding lengths along with observations regarding their respective stabilities. Stability in this context is the subjective judgement of whether the results are too stochastic, underfitted, or properly stable. It should be noted that once the minimum and maximum window sizes are found, meaning the ω_t values that produce too much stochasticity and underfitting, respectively, then exceeding those limits will only make output worse. There will be a single optimal window size or interval that produces the most stable, distinguishable output.

Table 1: All tested QtAC parameter combinations and their respective degrees of observed stability.

**Window Size** (ω_t)	**Embedding Length** (k, l)	**Stability** (Qualitative)
10	4, 10, 20	Stochastic
13	7, 8	Stochastic
16	8, 9	Stochastic
18	10, 11, 12, 13	Stochastic
20	10, 11, 12, 13, 14, 15	Stability Reached

23	10, 11, 12, 13, 14, 15	Stability Reached
25	4, 7, 10, 11, 12, 13, 14, 15, 20	Stability Reached

Higher embedding length values where stability was reached (k, l = 12, 13) were not testable with window sizes of the same magnitude or less because all datapoints would be considered in the kernel density estimation process, leading to underfitted results. This is why smaller window sizes had smaller embedding lengths which would statistically produce stochastic results more often than larger window sizes. It is a matter of how many data are available to the kernel density estimator: If less data are provided in a window and fewer nearest neighbors are considered, then the probability density estimation will be too stochastic. For all tests conducted in this study, the QtAC delay parameters were left at default values because there was no reason to alter them to better suit the properties of the Samothraki system.

Ultimately, the window size was increased to 25 where a distinguishable long-term trend was identified in multiple window size and embedding length combinations. At a window size of 20, the program output began to show two time periods where capacity to develop and ascendancy undergo gradual increases and decreases. The first period roughly spans 1960 – 1980, and the second period spans 1990 – 2002, approximately. Each test with a different window size and embedding length combination varied by sometimes several years in terms of when these two periods start and finish. However, three tested window sizes (ω_t = 20, 23, 25) exhibit this similar behavior even when varying their respective embedding lengths. Varying the embedding lengths changed the overall shape of the output's curves, resembling the changing probability densities due to the increased (greater k, l value) or decreased (lesser k, l value)

dependence on previous states. Figure 4 shows what is considered the best results obtained in this study (ω_t = 23, 25).

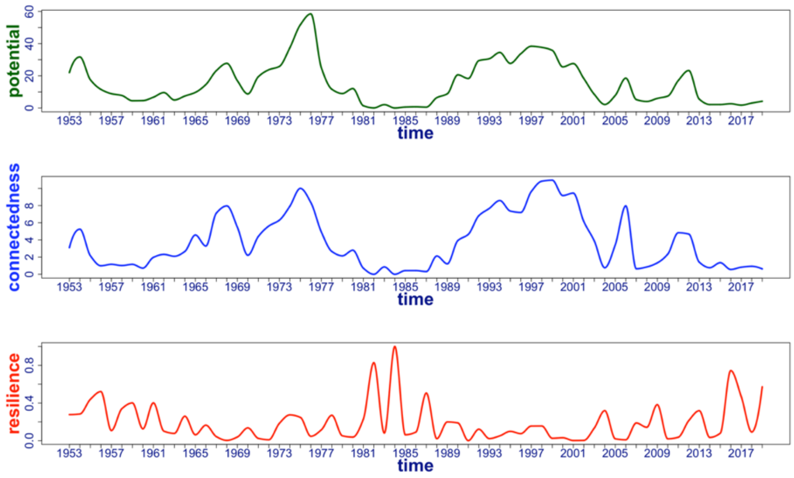

(a)

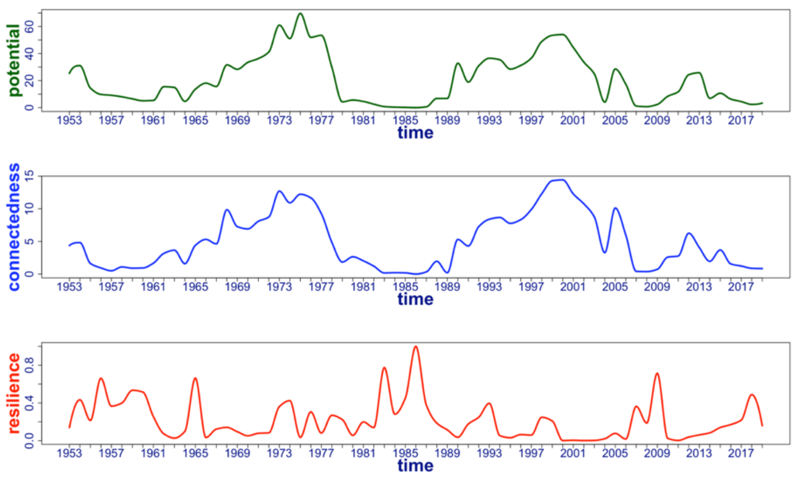

(b)

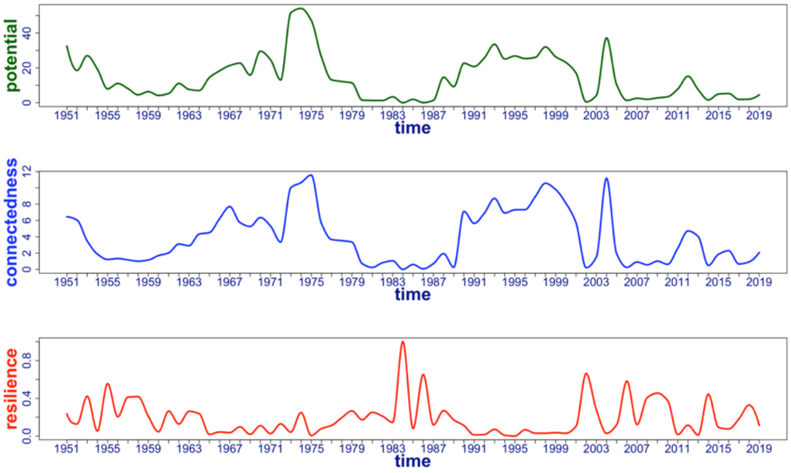

(c)

Figure 4: Example tests of the study's optimal results. Ascendancy analysis measures of Samothraki with (a) ω_t = 25 and *k, l* = 13, (b) ω_t = 25 and *k, l* = 12, and (c) ω_t = 23 and *k, l* = 12 (this study's chosen results for further analysis).

The numerical results from Figure 4 (c) were taken to be the single set of information-based ascendancy analysis output that were compared to the flow-based ascendancy analysis. Results from Figure 4 (a – b) could have been used as well – the goal of identifying evolutionary drivers of the Samothraki system can be accomplished from the measures' macroscopic behavior (i.e., highly local deviations/variations between all four datasets in Figure 4 are negligible for the study's purposes). Therefore, there were several $\omega_t/k/l$ parameter options that could have been used to execute the system analysis. The QtAC method does not, in the most deterministically rigorous sense, determine one optimal combination of parameters that should be analyzed. There is a tradeoff in stability and stochasticity that is left to the user's judgement.

If users are interested in a detailed explanation of the kernel density estimation process utilizing the KSG estimator, see references 18 and 21. The QtAC parameters used to obtain the best results are tabulated below.

Table 2: QtAC program parameters used to obtain the best ascendancy analysis results.

Parameter	Description
ω_t	23
k	12
l	12
k_tau	1 (default)
l_tau	1 (default)
$delay$	1 (default)
